# Synthesis and investigation of quadruplex-DNA-binding, 9-*O*-substituted berberine derivatives

**DOI:** 10.3762/bjoc.16.230

**Published:** 2020-11-18

**Authors:** Jonas Becher, Daria V Berdnikova, Heiko Ihmels, Christopher Stremmel

**Affiliations:** 1 Department of Chemistry and Biology, University of Siegen and Center of Micro- and Nanochemistry and Engineering (Cμ); Adolf-Reichwein-Str. 2, 57068 Siegen, Germany

**Keywords:** berberine alkaloids, DNA ligands, DNA recognition, G4-DNA, nucleic acids

## Abstract

A small series of five novel berberine derivatives was synthesized by the Cu-catalyzed click reaction of 9-propargyladenine with 9-*O*-(azidoalkyl)berberine derivatives. The association of the resulting berberine–adenine conjugates with representative quadruplex-forming oligonucleotides **22AG** dA(G_3_TTA)_3_G_3_ and **a2** d(ACAG_4_TGTG_4_)_2_ was examined with photometric and fluorimetric titrations, thermal DNA denaturation analysis, and CD spectroscopy. The results from the spectrometric titrations indicated the formation of 2:1 or 1:1 complexes (ligand:G4-DNA) with log *K*_b_ values of 10–11 (2:1) and 5–6 (1:1), which are typical for berberine derivatives. Notably, a clear relationship between the binding affinity of the ligands with the length of the alkyl linker chain, *n*, was not observed. However, depending on the structure, the ligands exhibited different effects when bound to the G4-DNA, such as fluorescent light-up effects and formation of ICD bands, which are mostly pronounced with a linker length of *n* = 4 (with **a2**) and *n* = 5 (with **22AG**), thus indicating that each ligand–G4-DNA complex has a specific structure with respect to relative alignment and conformational flexibility of the ligand in the binding site. It was shown exemplarily with one representative ligand from the series that such berberine–adenine conjugates exhibit a selective binding, specifically a selectivity to quadruplex DNA in competition with duplex DNA, and a preferential thermal stabilization of the G4-DNA forms **22AG** and **KRAS**. Notably, the experimental data do not provide evidence for a significant effect of the adenine unit on the binding affinity of the ligands, for example, by additional association with the loops, presumably because the adenine residue is sterically shielded by the neighboring triazole unit.

## Introduction

**Scheme 1:**
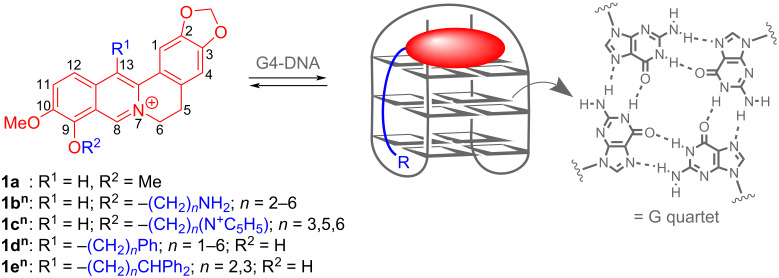
The structures and numbering of berberine (**1a**) and the alkyl-substituted derivatives **1a^n^**–**e^n^** and the binding equilibrium with quadruplex DNA (G4-DNA).

In nucleic acids chemistry, quadruplex DNA (G4-DNA) has been established as an attractive target [1–3]. This noncanonical DNA form is assembled through stacking of at least two guanine quartets and has been observed with highly diverse variation of structures in guanine-rich DNA sequences [4–6], for example, in the promoter regions of oncogenes or in single-stranded overhang of telomeric DNA [7–9]. Most notably, it has been shown that quadruplex formation is directly involved in biologically relevant processes [10], for example, in the suppression of gene expression [11–12] or the induction of the cellular response to DNA damage [13–14]. Because of the increasing evidence of an essential biological function of G4-DNA, this DNA form is considered as an attractive target in drug development [1–2,15–16]. For that purpose, G4-DNA-targeting ligands are searched for that bind selectively and sufficiently strong to quadruplex DNA and thereby influence the biological function of G-rich DNA sequences [17–22]. Among the numerous classes of compounds, mostly related to traditional DNA binders, that have been successfully developed as G4-DNA ligands [17], the natural product berberine (**1a**) has attracted special attention. Berberine (**1a**) is an isoquinoline alkaloid with an exceptionally wide range of biological activities [23–24]. It has been shown that berberine (**1a**) and its derivatives act, for example, as anti-inflammatory [25], antibacterial [26–27], and anticancer reagents [28–29]. The latter property is mainly based on the binding interaction of berberine with nucleic acids and the resulting inhibition of topoisomerase and telomerase [2,30]. Most notably, berberine (**1a**) induces a strong growth inhibition in several human cancer cells, but has only a relatively low cytotoxicity in healthy cells [31–32]. Berberine is also known as a G4-DNA ligand [33]. Especially berberine derivatives that carry additional substituents with varying alkyl chain lengths in the 9- and 13-position show enhanced binding properties and high selectivity towards telomeric G-quadruplex DNA [34–37]. Representative examples of this class of compounds are the 9-*O*-aminoalkyl-substituted and 9-*O*-pyridinium-*N*-alkyl-substituted derivatives **1b^n^** and **1c^n^** or the 13-phenylalkyl-substituted substrates **1d^n^** or **1e^n^** (Scheme 1) [38–42]. In the latter cases, the binding properties depend on the length of the alkyl chain. For example, the aminohexyl-substituted derivative **1b^6^** and the phenylpropyl-substituted compound **1d^3^** have the highest affinity to G-quadruplex DNA, whereas the derivatives with other alkyl chain lengths have a lower affinity [38]. Along the same line, the influence of the length and substituents of the side chains at the G4-DNA ligands have been assessed for quinolinium [43], indoloquinoline [44–45], phenanthroline [46], phenothiazine [47], and thiazole orange [48] derivatives. In these studies, the delicate balance between the hydrophobic effects of the alkyl chain and the thermodynamically favorable interactions on the association of ammonium or pyridinium groups in the grooves and loops was assessed. In another approach with a cyanine-based ligand, the alkyl substituents with a suitable length were terminated with an *N*-benzylamide functionality to establish the attractive hydrogen bonding and π stacking with the thymidine residues in the loops in G4-DNA, so that this ligand binds with very high selectivity to the particular quadruplex-forming oligonucleotide **J19** [49].

Overall, the above-mentioned observations indicate that berberine is among the more promising lead structures for the development of G4-DNA ligands. Moreover, the systematic variation of functional side-chains appears to be a suitable approach to determine the factors that influence the selectivity and affinity of a given ligand system. With this background, we proposed that the functionalization of the berberine scaffold with adenine-appended alkyl substituents may provide a useful platform to further explore this important aspect. Specifically, we wished to examine whether adenine–berberine conjugates with a varying linker length may allow to deduce a relationship between the chain length and the binding properties. The adenine unit was supposed to establish binding interactions with the loop region of the quadruplex, namely through Watson–Crick base pairing with the complementary thymidine residues. Herein, we describe the synthesis and characterization of the novel berberine–adenine conjugates **4a**–**e** along with the preliminary investigations of the interactions with selected G4-DNA forms, mainly **22AG** as the representative telomeric DNA sequence that may be considered a well-established reference, and **a2**, i.e., a quadruplex-forming repeat unit from the “insulin-linked polymorphic region” (ILPR) [50], that was also shown to bind quadruplex ligands [51].

## Results

### Synthesis

**Scheme 2:**
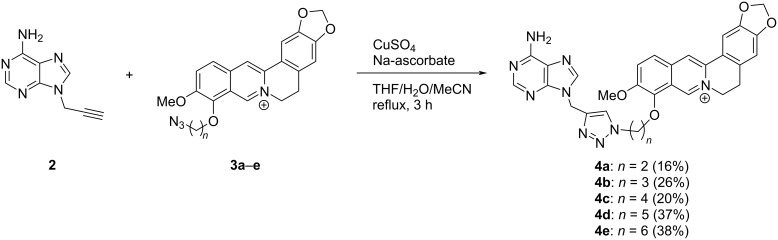
Synthesis of the berberine–adenine conjugates **4a**–**e**.

As the Cu-catalyzed click reaction between azides and alkynes is a well-established method for the variable functionalization of G4-DNA ligands [52], the berberine–adenine conjugates **4a**–**e** were synthesized by the reaction of 9-propargyladenine (**2**) [53] with the 9-azidoalkylberberine derivatives **3a**–**e** [54] (Scheme 2). Although the compounds **4a**–**e** formed as the major products in this reaction (>>50%), they were only obtained as isolated products in low to moderate yields (16–38%), mainly because of severe difficulties to completely remove the copper ions that apparently bind tightly to the compounds. The new compounds **4a**–**e** were identified and fully characterized with NMR spectroscopy (^1^H, ^13^C, COSY, HSQC, HMBC), mass spectrometry, and elemental analysis.

### DNA-binding properties

#### Spectrometric titrations

**Figure 1:**
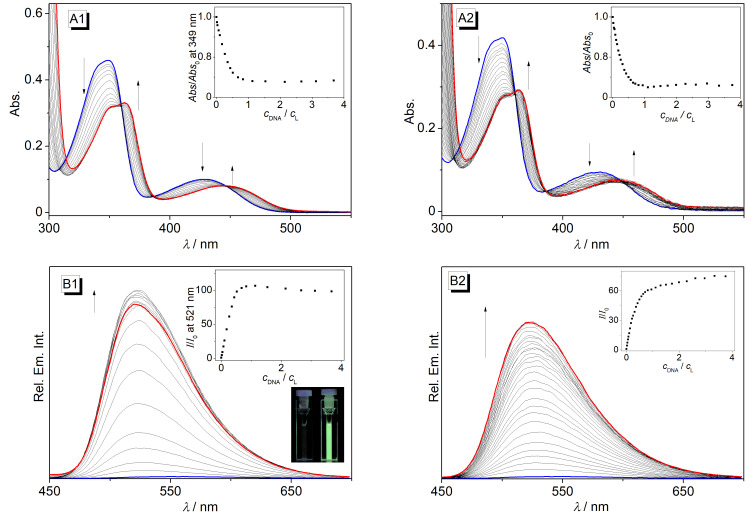
Representative spectrophotometric (A) and spectrofluorimetric (B) titration of compound **4c** with **22AG** (1) and **a2** (2) (*c***_3c_** = 20 μM; *c*_DNA_ = 200 μM) in K^+^-phosphate buffer (pH 7.0) with 10% v/v DMSO). The arrows indicate the development of the absorption or emission bands with increasing DNA concentration. Insets: Plots of *Abs.*/*Abs.*_0_ and *I*/*I*_0_, respectively, versus *c*_DNA_/*c***_3c_**. Inset B1: Picture of the emission color and intensity of compound **4c** in the absence (left) and the presence (right) of **22AG**.

The interactions of the conjugates **4a**–**e** with the quadruplex-forming oligonucleotides **22AG** dA(G_3_TTA)_3_G_3_ and **a2** d(ACAG_4_TGTG_4_)_2_ were analyzed by photometric and fluorimetric titrations (Figure 1). In all cases, the initial absorption maxima of the ligands **3a**–**e** decreased upon the addition of **22AG** or **a2** and new, slightly red-shifted absorption bands developed (Figure 1A; cf. Supporting Information File 1, Figure S1). During most of the titrations, the formation of an isosbestic point was observed. However, in several cases it clearly faded away at the end of the titration.

The compounds **4a**–**e** have a very low intrinsic emission intensity that increased significantly upon the addition of the G4-DNA **22AG** and **a2** (Table 1, Figure 1B; cf. Supporting Information File 1, Figure S2). Thus, the characteristic emission band of berberine at 520 nm developed and the relative intensity, *I*/*I*_o_, increased by factors ranging between 21 and 20 for **4b** and 71 and 107 for **4c**. This light-up effect can be easily followed by the naked eye (Figure 1B1, inset).

**Table 1:** The binding constants, *K*_b_, fluorescence light-up factors, *I*/*I*_0_*, and* shifts of the melting temperature, Δ*T*_m_, of compounds **4a**–**e** with G4-DNA.

	log *K*_b_^a^ (ligand/DNA)	*I*/*I*_0_^b^		log *K*_b_^a^ (ligand/DNA)	*I*/*I*_0_^b^		Δ*T*_m_ [°C]^c^		Δ*T*_m_ [°C]^c^
							
	**22AG**			**a2**			**F21T**		**Fa2T**

**4a**	5.89 ± 0.07 (1:1)	39		5.27 ± 0.08 (1:1), 10.3 ± 0.1 (2:1)	58		3.4		−0.5
**4b**	5.64 ± 0.15 (1:1), 10.8 ± 0.2 (2:1)	21		5.39 ± 0.14 (1:1), 10.6 ± 0.2 (2:1)	20		6.2		0.9
**4c**	5.23 ± 0.09 (1:1), 10.7 ± 0.1 (2:1)	107		10.6 ± 0.3 (2:1)	71		6.4		−0.1
**4d**	5.09 ± 0.13 (1:1), 10.8 ± 0.1 (2:1)	94		11.1 ± 0.2 (2:1)	52		9.9		0.0
**4e**	10.7 ± 0.1 (2:1)	29		11.1 ± 0.1 (2:1)	35		12.9		1.1

^a^Determined from the analysis of the photometric titration data with Specfit/32^TM^ with the adequate fits for complexes with ligand:DNA ratio 1:1 and 2:1. *K* in M^−1^ for 1:1 complexes and M^−2^ for 1:2 complexes. ^b^Determined from the fluorimetric titrations. ^c^Determined from the fluorimetric FRET experiment of **F21T** or **Fa2T** (*c*_DNA_ = 0.2 µM) at *LDR* 5.0 in Na-cacodylate buffer [*c*(K^+^) = 10 mM, pH 7.2]; *LDR* = 5; estimated error ± 0.5 °C.

The data from the photometric titrations were used to construct the corresponding binding isotherms and to determine the binding constants, *K*_b_, of **4a**–**e** with G4-DNA **22AG** and **a2** (cf. Supporting Information File 1). As a general trend, the experimental data could be adequately fitted to a binding stoichiometry ligand/G4-DNA of 2:1 or 1:1. Except for compound **4a**, all ligands formed 2:1 complexes with **22AG** and **a2**. The complexes of ligands **4b**–**e** with **22AG** have essentially the same log *K*_b_ values at 10.7–10.8 (*K*_b_ in M^−2^), whereas the log *K*_b_ values of ligands **4a**–**e** with **a2** increase slightly in the 2:1 complexes from 10.3 to 11.1 with increasing chain length *n* (Table 1). At the same time, 1:1 complexes were found for ligands **4a**–**d** and **22AG** as well as for ligands **4a**,**b** and **a2** with log *K*_b_ values between 5.1 (**4d** and **22AG**) and 5.9 (**4a** and **22AG**).

#### Thermal DNA denaturation analysis

**Figure 2:**
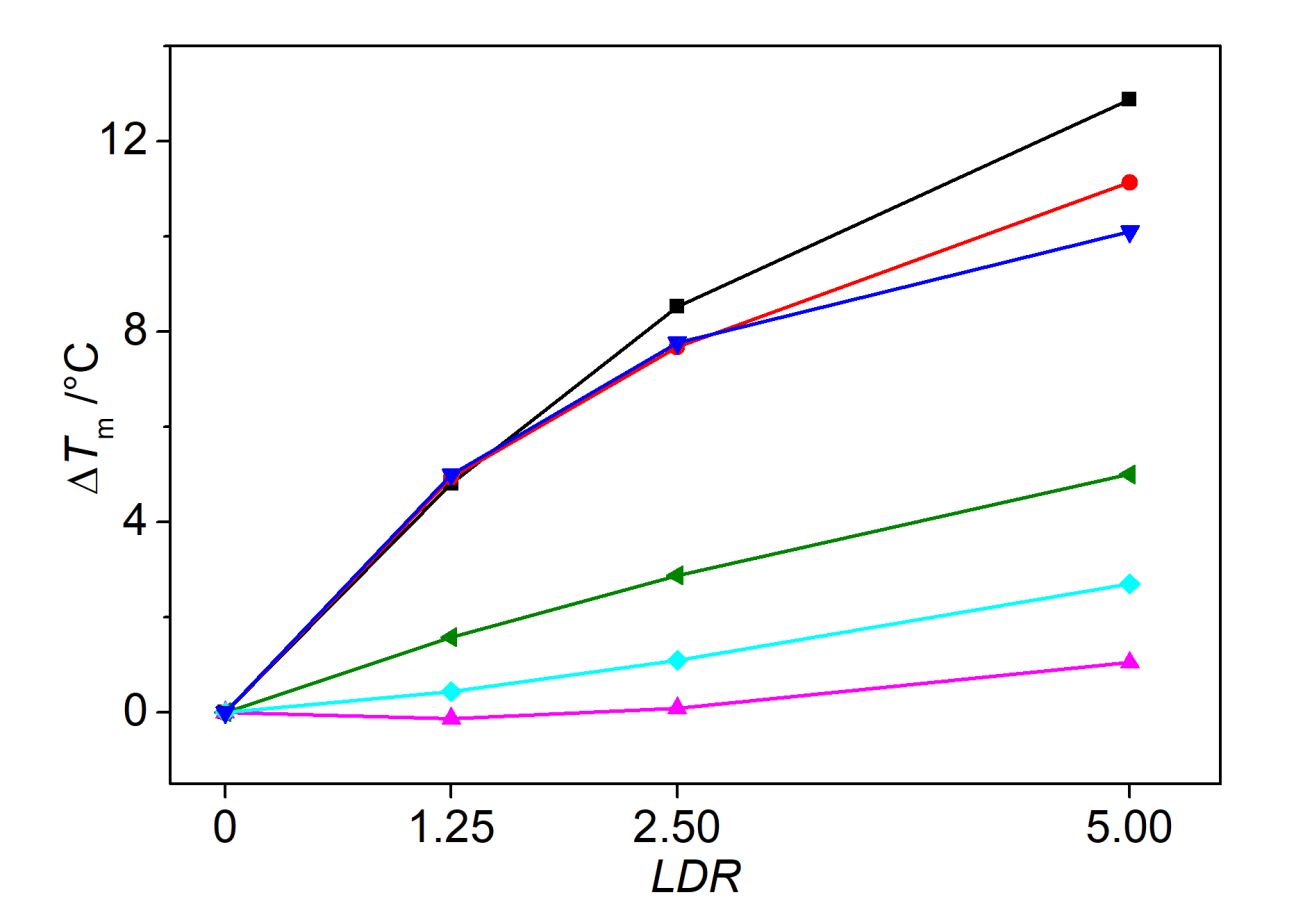
Melting temperatures, Δ*T_m_,* of G4-DNA (*c*_DNA_ = 0.2 µM) **F21T** (black), **F21T** plus **ds26** (15 equiv, red), **FkrasT** (blue), **FmycT** (green), **FkitT** (light blue), and **Fa2T** (magenta) in the presence of **4e** at different *LDR* = 0.00, 1.25, 2.50, 5.00 in Na-cacodylate buffer (*c*_K+_ = 10 mM, pH 7.2); estimated error ±0.5 °C. **F21T:** fluo-d[(G_3_TTA)_3_G_3_]-tamra; **Fa2T**: fluo-d[(ACAG_4_TGTG_4_)_2_-tamra; **FmycT**: fluo-d(TGAG_3_TG_3_TAG_3_TG_3_TA_2_)-tamra; **FkrasT**: fluo-d(AG_3_CG_2_TGTG_3_A_2_GAG_2_A)-tamra]; **FkitT**: fluo-d(AG_3_AG_3_CGCTG_3_AG_2_AG_3_)-tamra], fluo = fluorescein, tamra = tetramethylrhodamine; **ds26**: d(CA_2_TCG_2_ATCGA_2_T_2_CGATC_2_GAT_2_G).

In addition, the thermal stabilization of the G4-DNA **22AG** and **a2** upon the binding of the ligands **4a**–**e** was investigated by thermal DNA denaturation experiments. For that purpose, the DNA melting temperature *T*_m_ of the dye-labeled oligonucleotides **F21T** and **Fa2T** (for sequence see caption of Figure 2) was monitored by fluorescence spectroscopy, as the thermally induced unfolding of the quadruplex disrupts the Förster resonance energy transfer (FRET) between the two dyes. With this assay, the thermodynamic stabilization or destabilization of the quadruplex structure upon the complexation of the ligand is indicated by the shift of the melting temperature Δ*T*_m_*.* The analysis revealed an increasing stabilization of the quadruplex **F21T** toward dissociation with rising concentration of the ligand and with increasing chain length *n* of the ligands **4a**–**e**, as indicated by the shifts of the melting temperature of up to Δ*T*_m_ = 12.9 °C (Table 1). In contrast, the oligonucleotide **Fa2T** is only stabilized to a negligible extent upon the association of the ligands **4a**–**e** (Table 1; cf. Supporting Information File 1, Figure S4). In addition, it was examined exemplarily with the derivative **4e** whether the ligand also stabilizes other G4-DNA forms with different topologies. For that purpose, the representative quadruplex-forming oligonucleotides **FmycT**, **FkitT**, and **FkrasT** were also submitted to the thermal DNA denaturation experiments in the presence of **4e** (Figure 2; cf. Supporting Information File 1, Figure S5). In all cases, the quadruplex structure is stabilized by the ligand, but it was also observed that the degree of the G4-DNA stabilization upon the binding of the ligand **4e** is not the same for the different oligonucleotides as shown by the significantly different Δ*T*_m_ values. Specifically, the stabilization decreased in the order **F21T** (Δ*T*_m_ = 12.9 °C) > **FkrasT** (Δ*T*_m_ = 10.1 °C) > **FmycT** (Δ*T*_m_ = 5.0 °C) > **FkitT** (Δ*T*_m_ = 2.7 °C) > **Fa2T** (Δ*T*_m_ = 1.1 °C; at *LDR* = 5). Furthermore, the selectivity of the ligand **4e** toward quadruplex DNA in competition with duplex DNA was investigated by DNA denaturation experiments in the presence of an excess of the duplex-DNA forming oligonucleotide **ds26**. Under these conditions, the ligand **4e** shows essentially the same stabilization as in the absence of **ds26** as clearly indicated by only a small decrease of the melting temperature of ΔΔ*T*_m_ = 1.8 °C (Figure 2; cf. Supporting Information File 1, Figure S4).

#### CD spectroscopy

**Figure 3:**
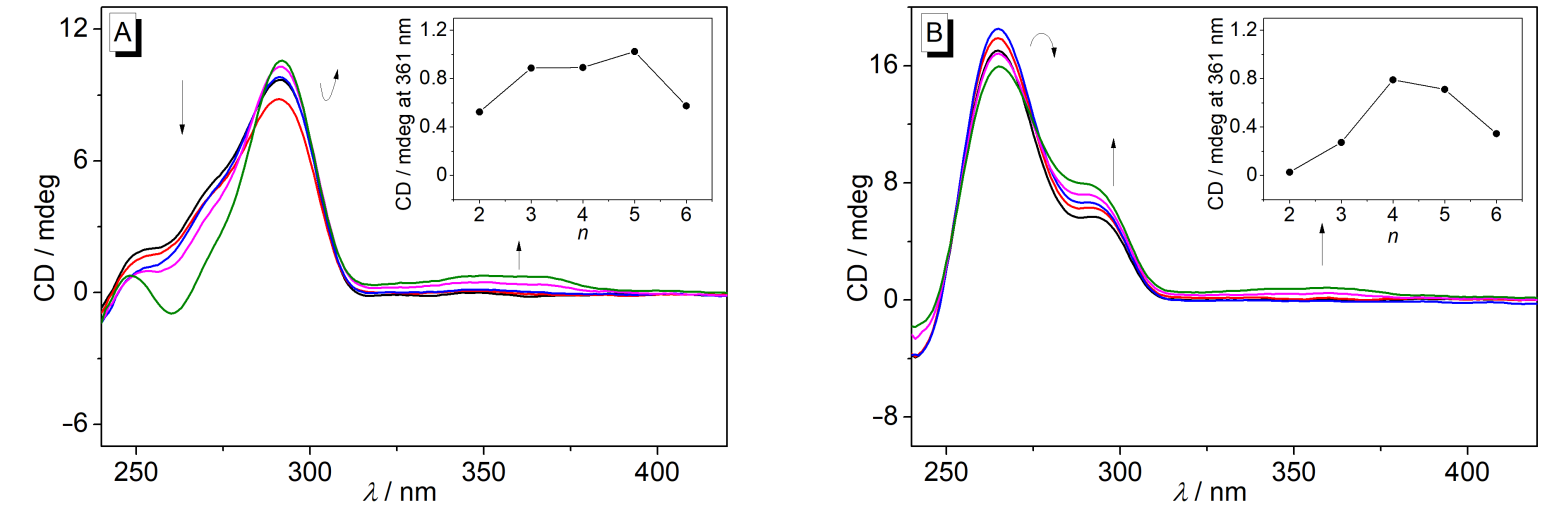
CD spectra of **22AG** (A) and **a2** (B) in the presence of the ligands **4c** (in K^+^-phosphate buffer (pH 7.0 with 10% v/v DMSO) at *LDR* 0.00 (black), 0.05 (red), 0.20 (blue), 0.50 (magenta), and 1.00 (green); *c*_DNA_ = 20 µM; *T* = 20 °C. The arrows indicate the development of the CD bands with increasing *LDR*. Insets: Plots of intensity of the ICD signal of the ligand–DNA mixtures (LDR 1.0; 361 nm) for **22AG** (A) and **a2** (B) versus the alkyl chain lengths *n* of **4a**–**e**.

The interactions of the ligands **4a**–**e** with G4-DNA **22AG** and **a2** were also examined with circular dichroism (CD) spectroscopy. Upon the addition of the ligands to **22AG** the positive CD band of the DNA at 295 nm remained essentially unchanged, whilst the blue-shifted shoulder to this band disappeared and a negative signal at 260 nm formed, whose intensity depended on the chain length between the berberine and the adenine unit and was the strongest with a chain length of *n* = 5 (Figure 3). In the case of **a2**, the positive band of this G4-DNA at 265 nm showed only small fluctuations upon the interaction with the ligands, whereas the intensity of the broad red-shifted shoulder at 295 nm slightly increased at higher *LDR*. In addition, during all titrations a weak induced CD (ICD) signal was formed in the absorption region of the ligands, which was most pronounced for the ligands with linker lengths of *n* = 5 on the association with **22AG** and of *n* = 4 on the binding to **a2** (Figure 3).

## Discussion

The results from the spectrometric titrations with **22AG** and **a2** clearly revealed the association of the derivatives **4a**–**e** with G4-DNA (Figure 1). Specifically, the red shift and hypochromism of the absorption band along with the pronounced increase of the emission intensity are typical for quadruplex-bound ligands [17–22]. Moreover, the resulting binding constants are in the same range as the ones reported for the resembling derivatives [33–42]. Notably, the analysis of the binding isotherms revealed the formation of 2:1 or 1:1 complexes (ligand/G4-DNA) (Table 1), both of which are also well-established for berberines [33–42]. Based on these general similarities between the derivatives **4a**–**e** and the established quadruplex-binding berberine derivatives it is deduced that the berberine unit in **4a**–**e** binds like the latter ones to the G4-DNA by terminal π-stacking, either with one or two ligands per quadruplex unit, essentially depending on the ligand/DNA ratio in solution.

Although the binding constants of the ligands with **4a**–**e** with **22AG** and **a2** deviate marginally within the series, they all lie essentially in the same order of magnitude. Thus, clear relationships between the length of the alkyl chain *n* and the binding constant *K*_b_ cannot be deduced from these data, as has been done with other alkyl-substituted berberine derivatives [38–42]. In the latter cases, however, the alkyl chains were substituted with positively charged functionalities that contributed significantly to the binding affinity depending on their spacing from the π-stacking unit. In the case of **4a**–**e**, however, the position of the triazole and adenine unit relative to the berberine does not appear to be highly relevant for the overall binding affinity. It may be concluded that the additional hydrophobic effect is the main contribution of the different substituents of **4a**–**e** to the overall binding affinity.

It is well known that the emission of the parent berberine increases strongly upon the accommodation in sterically constrained binding sites in, e.g., nucleic acids, cucurbiturils, cyclodextrins or micelles [55–58]. Presumably the radiationless deactivation of the excited state by conformational changes, that leads to the low emission intensity in aqueous solution, is suppressed in the sterically restricted binding site. Therefore, it can be deduced that the increased emission of the ligands **4a**–**e** on the addition of G4-DNA is the result of a sufficiently tight complexation. As this fluorescence light-up effect depends significantly on the length of the linker chain *n*, it is also concluded that the ligands with the strongest effect, i.e., **4c** and **4d** (*n* = 4 and 5), have a more restricted molecular flexibility in the binding pocket than the ones with shorter or longer side chains. It should be noted, however, that this binding mode does not lead to a significantly stronger binding affinity as the binding constants of **4c** with **22AG** or **a2** are only slightly different and even smaller than the ones of the other ligands (Table 1).

Obviously, the shifts of the melting temperature *ΔT*_m_ of G4-DNA in the presence of the ligands do not correlate well with the binding constants. Specifically, none of the ligands stabilizes the quadruplex **a2** towards unfolding, as is clearly shown by the negligible shifts of the melting temperatures, whereas the binding constants are in the dimension of 10^5^ M^−1^. In this context, it has to be emphasized that the binding constants *K*_b_ are not directly related to the ligand-induced shifts of the DNA melting temperature *T*_m_, because the latter also depends on other parameters such as binding-site size, cooperativity between ligands, ionic strength, the enthalpy of the DNA denaturation, and on the binding constant and enthalpy of the ligand binding at the melting temperature. However, the binding constant is determined at temperatures below *T*_m_ and the enthalpy of the ligand binding (Δ*H*_b_) is hardly accessible. Hence, we explain the very low *ΔT*_m_ values for G4-DNA **a2** in the presence of the ligands **4a**–**e** with a very low affinity of these ligands at the melting temperature, which may be caused by the delicate, temperature-dependent equilibrium of the different quadruplex forms of this particular DNA [50–51]. In the case of the G4-DNA **22AG**, the stabilization by the ligands **4a**–**e** is more consistent with the binding constants, as both sets of data indicate a moderately high binding constant and a good stabilization towards thermally induced unfolding (Table 1). Nevertheless, the binding constants do not deviate significantly in the series of ligands whereas the thermal stabilization is significantly more pronounced with the ligands **4c** and **4d**, which may indicate that these ligands have a slightly larger binding affinity to the DNA at the melting temperature than the other ones.

Most notably, the representative DNA denaturation analysis with ligand **4e** and different quadruplex forms clearly reveals a significant selectivity. Hence, the hybrid antiparallel G4-DNA **22AG** as well as the parallel quadruplex-forming **KRAS** sequence are stabilized to a significantly more extent than the parallel **c-kit**, **c-myc** or the mixed parallel/antiparallel **a2** sequence. Therefore, it is concluded that the selectivity of the ligands does not depend on the direction of the strands, i.e., parallel versus antiparallel, but on the loop structure of the respective quadruplex form. In particular, the G4-DNA **22AG** and **KRAS** apparently provide a suitable combination of accessibility of the terminal quartet for π-stacking with a loop structure that enables a favorable accommodation of the side chains. Although it may be assumed that additional interactions of the adenine residue with the loops assist the binding to the loops, there is no clear experimental evidence for this binding mode. This observation is in contrast to the report about an arylalkyl-substituted cyanine dye, that has been shown to bind with a high selectivity to particular G4-DNA forms because of additional attractive interactions with the loops [49]. In the latter case, however, the quadruplex-binding cyanine unit has been proposed to bind in the groove. In this binding mode, the alkyl-appended aryl functionalities may reach the loops and establish additional binding interactions more efficiently than the substituents of terminally stacked quadruplex ligand such as **4a**–**e**.

Along with the selective stabilization of particular quadruplex forms, the DNA denaturation analysis with ligand **4e** also showed a high selectivity for the quadruplex stabilization relative to duplex DNA as is clearly shown by only a small decrease of quadruplex melting in the presence of **4e** and an excess of the potentially competitive duplex DNA **ds26**. Although this experiment was only performed exemplarily with the ligand **4e** it may be carefully deduced that this class of compounds has a significantly higher affinity to quadruplex DNA.

Additional information about the complex formation between the ligands and the quadruplex DNA forms **22AG** and **a2** was provided by CD spectroscopy. The changes of the CD spectrum of **22AG** upon the addition of derivatives **4a**–**e** clearly indicate a shift of the equilibrium between the different quadruplex forms of **22AG** that are formed in the K^+^-containing buffer solution [59]. In particular, the decrease of the positive shoulder around 270 nm shows the disappearance of the (3 + 1) conformer, to which this band is assigned [59], in favor of the basket-type antiparallel quadruplex structure, which is identified by the characteristic CD pattern with a strong positive band at 295 nm and a weak negative one at 260 nm [60]. These observations show that all ligands stabilize preferentially the basket-type quadruplex structure of **22AG**. In the case of the G4-DNA **a2** the addition of the ligands shifts the equilibrium between the parallel and antiparallel quadruplex form only slightly in favor of the parallel structure, as is shown by a small increase of the positive CD signal at 295 nm, that is assigned to the parallel quadruplex [61–62], along with a decrease of the CD signal of the antiparallel form at 265 nm (Figure 3).

**Figure 4:**
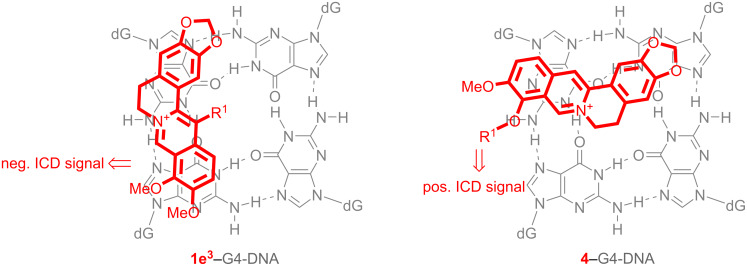
The simplified structure of the complex between **1e^3^** and quadruplex DNA (left; [38]) and the proposed orientation of the ligands **4a**–**e** with quadruplex DNA (right) according to ICD analysis (gray: G4 quartet; red: ligand).

Notably, weak, but significant ICD bands were observed in the absorption range of the ligands. Such ICD signals of DNA binders result from the dipole–dipole coupling of the ligands with the DNA bases and are typically observed for duplex DNA ligands [63–65]. In the case of the G4-DNA ligands, however, only very weak or even no ICD signals are often observed for the bound molecules, specifically for ligands that bind by terminal π-stacking. To the best of our knowledge, this phenomenon has not been discussed extensively in the literature, so far. As a result, clear relationships between the sign and pattern of the ICD signal of a quadruplex ligand in orientation relative to the binding site, as is well established for duplex binders [63], is not available for quadruplex ligands, yet. With most quadruplex-bound berberine and berberine derivatives, including compounds **1a**–**d**, ICD bands are not formed [34,36,40,66] or at least not explicitly mentioned; however, negative ICD bands [38,67] and exciton-type ICD signals [68] have been reported for some quadruplex-bound berberine derivatives. Unfortunately, in none of the latter cases the ICD signals were directly related to a particular binding mode. However, it was shown by X-ray diffraction analysis of the G4-DNA-bound ligand **1e^3^** that the berberine unit binds to quadruplex DNA by π-stacking with the 3’-end quartet in a similar mode as the parent berberine; however, in the case of **1e^3^** the aryl-substituents of the side chain are involved in the additional π-stacking with the G quartet (Figure 4) [38]. At the same time, the ligand **1e^3^** results a negative ICD when bound to quadruplex. Thus, considering that the derivatives **4a**–**e** have the same berberine fragment as binding unit and assuming that the phase of the ICD signal correlates directly with the relative alignment of the transition dipole moments of the ligand and the DNA bases [64], we carefully conclude that the positive ICD signal of the derivatives **4a**–**e** results from a binding mode in which the berberine unit is in a position essentially perpendicular to the one observed with **1e^3^** (Figure 4). In this structure the adenylalkyl substituent may be accommodated in the grooves or loops. The latter assumption is somehow supported by the observation that the intensity of the ICD signals varies depending on the chain lengths, indicating that the different fit of the side chain to the groove or loops has a direct influence on the strength and mode of the terminal π-stacking of the berberine unit. The tighter binding with better fitting of the side chain to the binding site is further supported by the observation that both ICD and the fluorescence light-up effect are the strongest with the chain length of 4 and 5 (Table 1).

Unfortunately, the experimental data do not provide any evidence for a relevant effect of the adenine unit on the binding affinity of the ligands **4a**–**e** or the complex structures with G4-DNA, because in this case significantly stronger differences of the binding constants, selectivities or optical responses should have been observed with the variation of the linker length. It may be concluded that the triazole ring, used as a synthetically convenient connection unit, imposes too much steric hindrance and restricted conformational flexibility in the vicinity of the adenine unit thus hindering the binding of the latter to the thymidine residues in the loops.

## Conclusion

In summary, we have synthesized five novel berberine–adenine derivatives **4a**–**e** with different lengths of the alkyltriazole linker units, which show the characteristic properties of berberine-based G4-DNA ligands. Notably, the binding affinities of the ligands do not change strongly with the length of the alkyl chain *n* and there is no obvious relationship between these parameters. Nevertheless, depending on the structure the ligands exhibit some significantly different effects when bound to the G4-DNA, such as fluorescent light-up effects and the formation of ICD bands, which are mostly pronounced with linker length of *n* = 4 (with **a2**) and *n* = 5 (with **22AG**). This significant influence of the complex structure on the optical properties of the ligand provides some evidence that each ligand–G4-DNA complex has a specific structure with respect to the relative alignment and conformational flexibility of the ligand in the binding site. Considering these changes upon variation of either the ligand structure or the quadruplex form, the ligands **4a**–**e** may have the potential to operate as selective ligands for G4-DNA, as indeed was shown exemplarily with the ligand **4e**. The latter has a high selectivity to quadruplex DNA in competition with duplex DNA and stabilizes preferentially the G4-DNA forms **22AG** and **KRAS**. We conclude from these results, along with the already reported data in the literature [36–37,39,42], that the derivatization of berberine by the attachment of functional substituents at the 9-position is a reasonable approach to fine-tune the binding properties with G4-DNA. However, more systematic investigations and broader structural variations are necessary to identify all relevant factors that affect the affinity and selectivity of such ligands.

## Experimental

### Equipment

NMR data were recorded with a Varian VNMR-S600 spectrometer [600 MHz (^1^H), 150 MHz (^13^C)] at 35 °C. NMR spectra were processed with the software ACD/NMR Processor Academic Version 12.01 and are referenced to the corresponding solvent [δ(DMSO-*d*_5_) = 2.50 (^1^H) and 39.5 (^13^C); δ(CHCl_3_) = 7.26 (^1^H) and 77.2 (^13^C)]. Elemental analyses data were determined with a HEKAtech EURO EA combustion analyzer by Mr. Rochus Breuer (Organic Chemistry I, University of Siegen). Mass spectra (ESI) were recorded on a Finnigan LCQ Deca (*U* = 6 kV; working gas: argon; auxiliary gas: nitrogen; temperature of the capillary: 200 °C). Absorption spectra were obtained with a Varian Cary 100 bio spectrometer in quartz cells (10 mm) with baseline correction. Emission spectra were recorded in quartz cells (10 mm) with a Cary Eclipse spectrometer at 20 °C. CD-spectroscopic measurements were performed with a Chirascan spectrometer (Applied Photophysics) in quartz cells (1.0 mm). Melting points were measured with a Büchi 545 (Büchi, Flawil, CH) and are uncorrected.

### Materials

Commercially available reagents and reactants were used without further purification. Chemicals were obtained from the following companies: Carl Roth GmbH & Co. KG (Karlsruhe, D): Berberine chloride, 3-bromo-1-propyne. Alfa Aesar GmbH & Co. KG (Karlsruhe, D): Adenine. Acros Organics: 3-Phenyl-1-propyne. Biomers.net GmbH (Ulm, D): **22AG**, **a2**, **F21T**, **Fa2T**, **FmycT**, **FkitT**, and **FkrasT**. 9-(Azidoalkyl)berberine derivatives **3a**–**e** [54] and 9-propargyladenine (**2**) [53] were synthesized according to published procedures. Stock solutions of the ligands (1.0 mM) were prepared in MeOH (HPLC grade). Buffer solutions were prepared with biochemical grade chemicals in E-Pure water (resistivity ≥ 18 MΩ cm) and filtered through a membrane filter (0.45 µm pore size). K-phosphate-buffer (**22AG**, **a2**): 25 mM K_2_HPO_4_, 70 mM KCl; pH 7.0; Na-cacodylate buffer (**F21T**, **Fa2T**, **FmycT**, **FkitT**, and **FkrasT**): 90 µM LiCl, 10 mM KCl, 10 mM Na(CH_3_)_2_AsO_2_ × 3H_2_O, pH 7.2–7.3. The K-phosphate buffer solutions and the Na-cacodylate buffer solutions were stored at 4 °C in the dark.

### Methods

The spectrophotometric and spectrofluorometric titrations with quadruplex DNA were performed according to published protocols [69]. To ensure a sufficient solubility during the titrations DMSO (1% v/v in BPE buffer and 5% v/v in K^+^-phosphate buffer) was used as a cosolvent.

For the CD spectra, solutions of G4-DNA in K^+^-phosphate butter and the ligands in buffer/DMSO were recorded after an equilibration time of 30 min.

For the thermal DNA denaturation analyses stock solutions of the ligand (20 µM) and the G4-DNA (50 µM) in Na-cacodylate buffer were used to prepare analyte solutions with different ligand:DNA ratios (*LDR* = 0.00, 1.25, 2.50, 5.00).

### Synthesis

#### General procedure (GP) [54]

To a solution of the berberine azide derivatives **3a**–**e** (1.0 molar equiv) and 9-propargyladenine (**2**, 1.1 molar equiv) in THF/MeCN 2:1 was added a solution of CuSO_4_ (0.3 equiv) and Na-ascorbate (1.1 molar equiv) in H_2_O. The mixture was stirred under reflux for 3 h. The solvent was removed under reduced pressure and the brown residue was dissolved in DMSO (15 mL) and filtered through a pad of neutral aluminum oxide. The pad was washed with DMSO (15 mL). The DMSO fractions were combined and the solvent was removed in vacuum. The remaining yellow solid was suspended in MeCN (500 mL) and filtered through a short pad of celite. The solvent was evaporated under reduced pressure and the crude product was purified by column chromatography (SiO_2_, CH_2_Cl_2_/MeOH 5→10%). After crystallization of the major fraction from MeOH/Et_2_O 7:3 the desired product was obtained as yellow solid.

#### 9-*O*-{β-{4'-[(9''-Adenyl)methyl]-1*H*-1',2',3'-triazol-1'-yl}ethyl}berberine bromide (**4a**)

According to the GP, a solution of **3a** (150 mg, 292 µmol), **2** (60.6 mg, 350 µmol), CuSO_4_ (5.83 mg, 36.5 µmol) and Na-ascorbate (36.3 mg, 183 µmol) was stirred at reflux in THF/H_2_O/MeCN 2:2:1 (25 mL). After purification by column chromatography (*R*_f_ = 0.11; 10% MeOH) and crystallization the product **4a** was obtained as orange-colored amorphous solid (30.0 mg, 46.5 µmol, 16%); mp 191–194 °C (dec.); ^1^H NMR (600 MHz, DMSO-*d*_6_) δ 3.20 (t, ^3^*J* = 6.5 Hz, 2H, 5-H), 3.94 (s, 3H, O-*CH*_3_), 4.70 (t, ^3^*J* = 5.0 Hz, 2H,α-H), 4.84 (t, ^3^*J* = 6.5 Hz, 2H, 6-H), 4.90 (t, ^3^*J* = 5.0 Hz, 2H, β-H), 5.44 (s, 2H, C-*CH*_2_-N) 6.18 (s, 2H, O-CH_2_-O), 7.09 (s, 1H, 4-H), 7.23 (s, 2H, NH_2_) 7.77 (s, 1H, 1-H), 7.93 (d, ^3^*J* = 9.2 Hz, 12-H), 8.04 (s, 1H, 2''-H), 8.10 (d, ^3^*J* = 9.2 Hz, 1H, 11-H), 8.19 (s, 1H, 8''-H), 8.30 (s, 1H, 5'-H), 8.89 (s, 1H, 13-H), 9.48 (s, 1H, 8-H); ^13^C NMR (150 MHz, DMSO-*d*_6_) δ 26.3 (C5), 38.0 (C-*CH*_2_-N), 50.0 (Cβ), 55.4 (C6), 57.0 (O-*CH*_3_), 72.1 (Cα), 102.1 (O-*CH*_2_-O), 105.5 (C1), 108.4 (C4), 118.5 (C5''), 120.2 (C13), 120.3 (C1a), 121.2 (C8a), 123.8 (C12), 124.2 (C5'), 126.4 (C11), 130.6 (C4a), 132.8 (C12a), 137.4 (C13a), 140.7 (C8''), 141.7 (C9), 143.0 (C4'), 145.0 (C8), 147.7 (C2), 149.2 (C4''), 149.9 (C3), 150.0 (C10), 152.5 (C2''), 155.9 (C6''); ESIMS (*m*/*z*): 564 [M^+^]; anal. calcd for C_29_H_26_BrN_9_O_4_ (%): C, 54.05; H, 4.07; N, 19.56; found (%): C, 54.03; H, 4.41; N, 19.44.

#### 9-*O*-{γ-{4'-[(9''-Adenyl)methyl]-1*H*-1',2',3'-triazol-1'-yl}propyl}berberine bromide (**4b**)

According to the GP, a solution of **3b** (120 mg, 247 µmol), **2** (60.6 mg, 350 µmol), CuSO_4_ (5.01 mg, 31.4 µmol), and Na-ascorbate (47.1 mg, 272 µmol) was stirred at reflux in THF/H_2_O/MeCN 2:2:1 (25 mL). After purification by column chromatography (*R*_f_ = 0.12, 10% MeOH) and crystallization the product **4b** was obtained as yellow, amorphous solid (42.3 mg, 64.0 µmol, 26%); mp 189–192 °C (dec.); ^1^H NMR (600 MHz, DMSO-*d*_6_) δ 2.41 (tt, ^3^*J* = 7 Hz, ^3^*J* = 7 Hz, 2H, β-H), 3.21 (t, ^3^*J* = 6 Hz, 2H, 5-H), 3.99 (s, 3H, O-*CH*_3_), 4.25 (t, ^3^*J* = 6 Hz, 2H, α-H), 4.67 (t, ^3^*J* = 7 Hz, 2H, γ-H), 4.94 (t, ^3^*J* = 6 Hz, 2H, 6-H), 5.45 (s, 2H, C-*CH*_2_-N), 6.18 (s, 2H, O-*CH*_2_-O), 7.09 (s, 1H, 4-H), 7.21 (s, 2H, *NH*_2_), 7.80 (s, 1H, 1-H), 7.99 (d, ^3^*J* = 9 Hz, 12-H), 8.08 (s, 1H, 2''-H), 8.16 (d, ^3^*J* = 9 Hz, 1H, 11-H), 8.21 (s, 1H, 8''-H), 8.24 (s, 1H, 5'-H), 8.94 (s, 1H, 13-H), 9.77 (s, 1H, 8-H); ^13^C NMR (150 MHz, DMSO-*d*_6_) δ 26.3 (5-C), 30.2 (Cβ), 38.1 (C-*CH*_2_-N), 46.6 (Cγ), 55.2 (C6), 57.0 (O-*CH*_3_), 71.7 (Cα), 102.1 (O-*CH*_2_-O), 105.5 (C1), 108.4 (C4), 118.5 (C5''), 120.2 (C13), 120.4 (C1a), 121.4 (C8a), 123.6 (C12), 123.8 (C5'), 126.6 (C11), 130.7 (C4a), 133.0 (C12a), 137.5 (C13a), 140.7 (C8''), 142.3 (C9), 142.7 (C4'), 145.2 (C8), 147.7 (C2), 149.3 (C4''), 149.8 (C3), 150.2 (C10), 152.5 (C2''), 155.9 (C6''); ESIMS (*m*/*z*): 578 [M^+^]; anal. calcd for C_30_H_28_BrN_9_O_4_ (%): C, 54.72; H, 4.29, N, 19.14; found (%): C, 54.69; H, 4.41; N, 18.79.

#### 9-*O*-{δ-{4'-[(9''-Adenyl)methyl]-1*H*-1',2',3'-triazol-1'-yl}butyl}berberine bromide (**4c**)

According to the GP, a solution of **3c** (240 mg, 481 µmol), **2** (96.1 mg, 529 µmol), CuSO_4_ (10.3 mg, 64.5 µmol), and Na-ascorbate (53.5 mg, 270 µmol) was stirred at reflux in THF/H_2_O/MeCN 2:2:1 (35 mL). After purification by column chromatography (*R*_f_ = 0.17, 10%) and crystallization the product **4c** was obtained as yellow, amorphous solid (64.9 mg, 96.5 µmol, 20%); mp 157–158 °C (dec.); ^1^H NMR (600 MHz, DMSO-*d*_6_) δ 1.81 (tt, ^3^*J* = 7 Hz, ^3^*J* = 7 Hz, 2H, β-H), 2.06 (tt, ^3^*J* = 7 Hz, ^3^*J* = 7 Hz, 2H, γ-H), 3.21 (t, ^3^*J* = 6 Hz, 2H, 5-H), 4.02 (s, 3H, O-*CH*_3_), 4.26 (t, ^3^*J* = 6 Hz, 2H, α-H), 4.46 (t, ^3^*J* = 7 Hz, 2H, δ-H), 4.93 (t, ^3^*J* = 6 Hz, 2H, 6-H), 5.44 (s, 2H, C-*CH*_2_-N), 6.18 (s, 2H, O-*CH*_2_-O), 7.10 (s, 1H, 4-H), 7.23 (s, 2H, *NH*_2_), 7.80 (s, 1H, 1-H), 7.98 (d, ^3^*J* = 9 Hz, 12-H), 8.12 (s, 1H, 2''-H), 8.15 (s, 1H, 5'-H), 8.17 (d, ^3^*J* = 9 Hz, 1H, 11-H), 8.20 (s, 1H, 8''-H), 8.93 (s, 1H, 13-H), 9.78 (s, 1H, 8-H); ^13^C NMR (150 MHz, DMSO-*d*_6_) δ 26.3 (Cγ), 26.3 (C5), 26.5 (Cβ), 38.0 (C-*CH*_2_-N), 49.1 (Cδ), 55.3 (C6), 57.0 (O-*CH*_3_), 73.4 (Cα), 102.1 (O-*CH*_2_-O), 105.4 (C1), 108.4 (C4), 118.5 (C5''), 120.2 (C13), 120.4 (C1a), 121.5 (C8a), 123.4 (C12), 123.6 (C5'), 126.6 (C11), 130.7 (C4a), 133.0 (C12a), 137.5 (C13a), 140.6 (C8''), 142.5 (C4'), 142.6 (C9), 145.2 (C8), 147.7 (C2), 149.3 (C4''), 150.3 (C3), 152.5 (C2''), 155.9 (C6''); ESIMS (*m*/*z*): 592 [M^+^]; anal. calcd for C_31_H_30_BrN_9_O_4_ × H_2_O (%): C, 53.92; H, 4.67; N, 18.26; found (%): C, 54.07; H, 4.98; N, 18.07.

#### 9-*O*-{ε-{4'-[(9''-Adenyl)methyl]-1*H*-1',2',3'-triazol-1'-yl}pentyl}berberine bromide (**4d**)

According to the GP, a solution of **3d** (150 mg, 292 µmol), **2** (60.6 mg, 350 µmol), CuSO_4_ (5.83 mg, 36.5 µmol), and Na-ascorbate (36.6 mg, 183 µmol) was stirred at reflux in THF/H_2_O/MeCN 2:2:1 (25 mL). After purification by column chromatography (*R*_f_ = 0.19, 10% MeOH) and crystallization the product **4d** was obtained as yellow, amorphous solid (73.2 mg, 107 µmol, 37%); mp 154–156 °C (dec.); ^1^H NMR (600 MHz, DMSO-*d*_6_) δ 1.41 (tt, ^3^*J* = 8 Hz, ^3^*J* = 7 Hz, 2H, γ-H), 1.86 (tt, ^3^*J* = 8 Hz, ^3^*J* = 7 Hz, 2H, β-H), 1.90 (tt, ^3^*J* = 7 Hz, ^3^*J* = 7 Hz, 2H, δ-H), 3.20 (t, ^3^*J* = 6 Hz, 2H, 5-H), 4.02 (s, 3H, O-*CH*_3_), 4.24 (t, ^3^*J* = 7 Hz, 2H, α-H), 4.39 (t, ^3^*J* = 7 Hz, 2H, ε-H), 4.95 (t, ^3^*J* = 6 Hz, 2H, 6-H), 5.42 (s, 2H, C-*CH*_2_-N), 6.17 (s, 2H, O-*CH*_2_-O), 7.08 (s, 1H, 4-H), 7.21 (s, 2H, *NH*_2_), 7.79 (s, 1H, 1-H), 7.98 (d, ^3^*J* = 9 Hz, 12-H), 8.11 (s, 1H, 2''-H), 8.14 (s, 1H, 5'-H), 8.16 (d, ^3^*J* = 9 Hz, 1H, 11-H), 8.19 (s, 1H, 8''-H), 8.94 (s, 1H, 13-H), 9.72 (s, 1H, 8-H); ^13^C NMR (150 MHz, DMSO-*d*_6_) δ 22.3 (Cγ), 26.3 (C5), 28.8 (Cβ), 29.4 (Cδ), 38.1 (C-*CH*_2_-N), 49.4 (Cε), 55.3 (C6), 57.0 (O-*CH*_3_), 73.9 (Cα), 102.1 (O-*CH*_2_-O), 105.5 (C1), 108.4 (C4), 118.6 (C5''), 120.3 (C13), 120.5 (C1a), 121.6 (C8a), 123.4 (C12), 123.5 (C5'), 126.6 (C11), 130.7 (C4a), 133.0 (C12a), 137.4 (C13a), 140.7 (C8''), 142.5 (C4'), 147.7 (C2), 149.3 (C4''), 149.8 (C3), 150.4 (C10), 152.5 (C2''), 155.9 (C6''); ESIMS (*m*/*z*): 606 [M^+^]; anal. calcd for C_32_H_32_BrN_9_O_4_ (%): C, 55.98; H, 4.70; N, 18.36; found (%): C, 55.70; H, 4.71; N, 18.11.

#### 9-*O*-{ζ-{4'-[(9''-Adenyl)methyl]-1*H*-1',2',3'-triazol-1'-yl}hexyl}berberine bromide (**4e**)

According to the GP, a solution of **3e** (300 mg, 567 µmol), **2** (118 mg, 682 µmol), CuSO_4_ (11.3 mg, 70.9 µmol), and Na-ascorbate (71.0 mg, 355 µmol) was stirred at reflux in THF/H_2_O/MeCN 2:2:1 (50 mL). After purification by column chromatography (*R*_f_ = 0.20, 10% MeOH) and crystallization the product **4e** was obtained as yellow, amorphous solid (151 mg, 216 µmol, 38%); mp 152–154 °C (dec.); ^1^H NMR (600 MHz, DMSO-*d*_6_) δ 1.27–1.32 (m, 2H, δ-H), 1.45–1.49 (m, 2H, γ-H), 1.80–1.85 (m, 4H, β-H, ε-H), 3.20 (t, ^3^*J* = 6.2 Hz, 2H, 5-H), 4.02 (s, 3H, O-*CH*_3_), 4.23 (t, ^3^*J* = 6.8 Hz, 2H, α-H), 4.35 (t, ^3^*J* = 7.0 Hz, 2H, ζ-H), 4.97 (t, ^3^*J* = 6.2 Hz, 2H, 6-H), 5.43 (s, 2H, C-*CH*_2_-N), 6.17 (s, 2H, O-*CH*_2_-O), 7.07 (s, 1H, 4-H), 7.22 (s, 2H, *NH*_2_), 7.78 (s, 1H, 1-H), 7.98 (d, ^3^*J* = 9.2 Hz, 12-H), 8.12 (s, 1H, 2''-H), 8.15 (s, 1H, 5'-H), 8.16 (d, ^3^*J* = 9.2 Hz, 1H, 11-H), 8.20 (s, 1H, 8''-H), 8.97 (s, 1H, 13-H), 9.75 (s, 1H, 8-H); ^13^C NMR (150 MHz, DMSO-*d*_6_) δ 24.6 (Cγ), 25.5 (Cβ), 26.3 (C5), 29.2 (Cδ), 29.6 (Cε), 38.0 (C-*CH*_2_-N), 50.0 (Cζ), 55.4 (C6), 57.0 (O-*CH*_3_), 72.1 (Cα), 102.1 (O-*CH*_2_-O), 105.5 (C1), 108.5 (C4), 118.5 (C5''), 120.2 (C13), 120.4 (C1a), 121.6 (C8a), 123.3 (C12), 123.5 (C5'), 126.6 (C11), 130.6 (C4a), 133.0 (C12a), 137.4 (C13a), 140.6 (C8''), 142.8 (C4'), 147.7 (C2), 149.2 (C4''), 149.9 (C3), 150.3 (C10), 152.5 (C2''), 155.9 (C6''); ESIMS (*m*/*z*): 620 [M^+^]; anal. calcd for C_32_H_34_BrN_9_O_4_ × 3H_2_O (%): C, 52.52; H, 5.34; N, 16.70; found (%): C, 52.54; H, 5.00; N, 16.39.

## Supporting Information

File 1 Experimental procedures, additional spectroscopic data, ^1^H NMR and ^13^C NMR spectra.
