# Supplementary material for: Synthesis and investigation of quadruplex-DNA-binding, 9-O-substituted berberine derivatives
Source: Beilstein J Org Chem. 2020 Nov 18;16:2795–806. doi: 10.3762/bjoc.16.230 (PMC7684686; doi:10.3762/bjoc.16.230)
Supplement: File 1 — Experimental procedures, additional spectroscopic data, 1H NMR and 13C NMR spectra. [file Beilstein_J_Org_Chem-16-2795-s001.pdf]

BEILSTEIN JOURNAL OF ORGANIC CHEMISTRY

Supporting Information

for

Synthesis and investigation of quadruplex-DNA-binding, 9-*O*-substituted berberine derivatives

Jonas Becher, Daria V. Berdnikova, Heiko Ihmels and Christopher Stremmel

Beilstein J. Org. Chem. **2020,** *16,* 2795–2806. doi:10.3762/bjoc.16.230

Experimental procedures, additional spectroscopic data, ^{1}H NMR and ^{13}C NMR spectra

License and Terms: This is a supporting information file under the terms of the Creative Commons Attribution License (https://creativecommons.org/licenses/by/4.0). Please note that the reuse, redistribution and reproduction in particular requires that the authors and source are credited.
The license is subject to the *Beilstein Journal of Organic Chemistry* terms and conditions: (https://www.beilstein-journals.org/bjoc)

Table of contents

1. Photometric and fluorimetric titrations S2
2. CD spectroscopy S6
3. Thermal DNA-denaturation S9
4. NMR spectra S11

1. Photometric and fluorimetric titrations

All DNA titrations with **22AG** or **a2** were performed in potassium phosphate buffer [$c(K^+)$ = 120 mM, pH 7.0; with 10% v/v DMSO]. The titrant solution contained the same ligand concentration (c_L) as the analyte solution to ensure a constant ligand concentration throughout the titration. The excitation wavelength in the fluorimetric titrations for all ligands was the isosbestic point around λ = 360 nm as obtained by the corresponding photometric titration. After recording the pure ligand solution, small aliquots of the DNA solution were titrated to the ligand solution until no significant change in the absorbance or fluorescence intensity was observed. The spectra were recorded after an equilibration time of 3 min (Figure S1 and Figure S2).

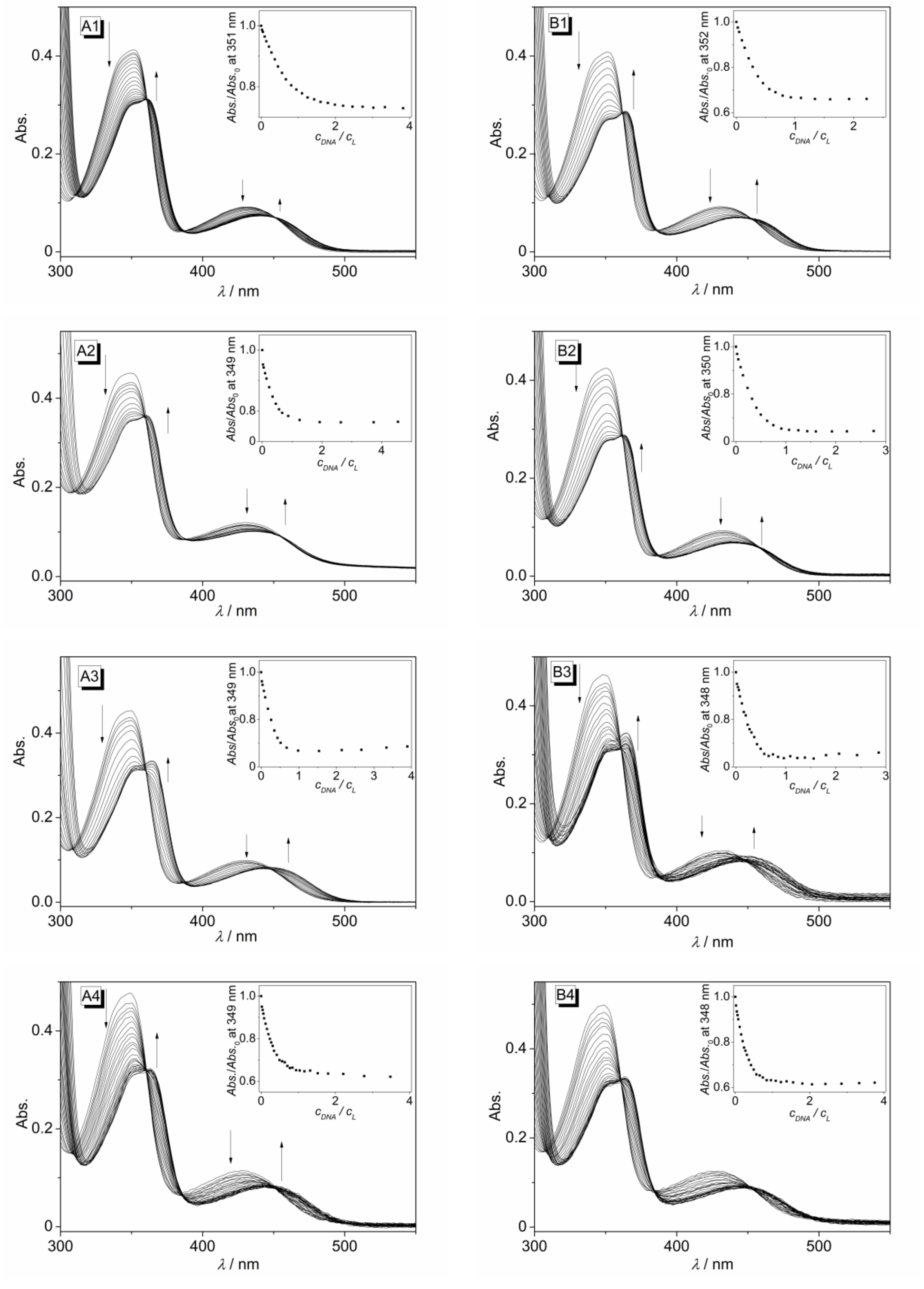

Figure S1. Photometric titration of **4a** (1), **4b** (2), **4d** (3) and **4e** (4) with **22AG** (A) and **a2** (B) (c_L = 20 µM; c_{DNA} = 200 µM) in K$^+$-phosphate buffer (pH 7.0 with 10% v/v DMSO). The arrows indicate the development of the absorption bands with increasing DNA concentration. Inset: plot of *Abs.*/*Abs.*$_0$ versus c_{DNA}/c_L.

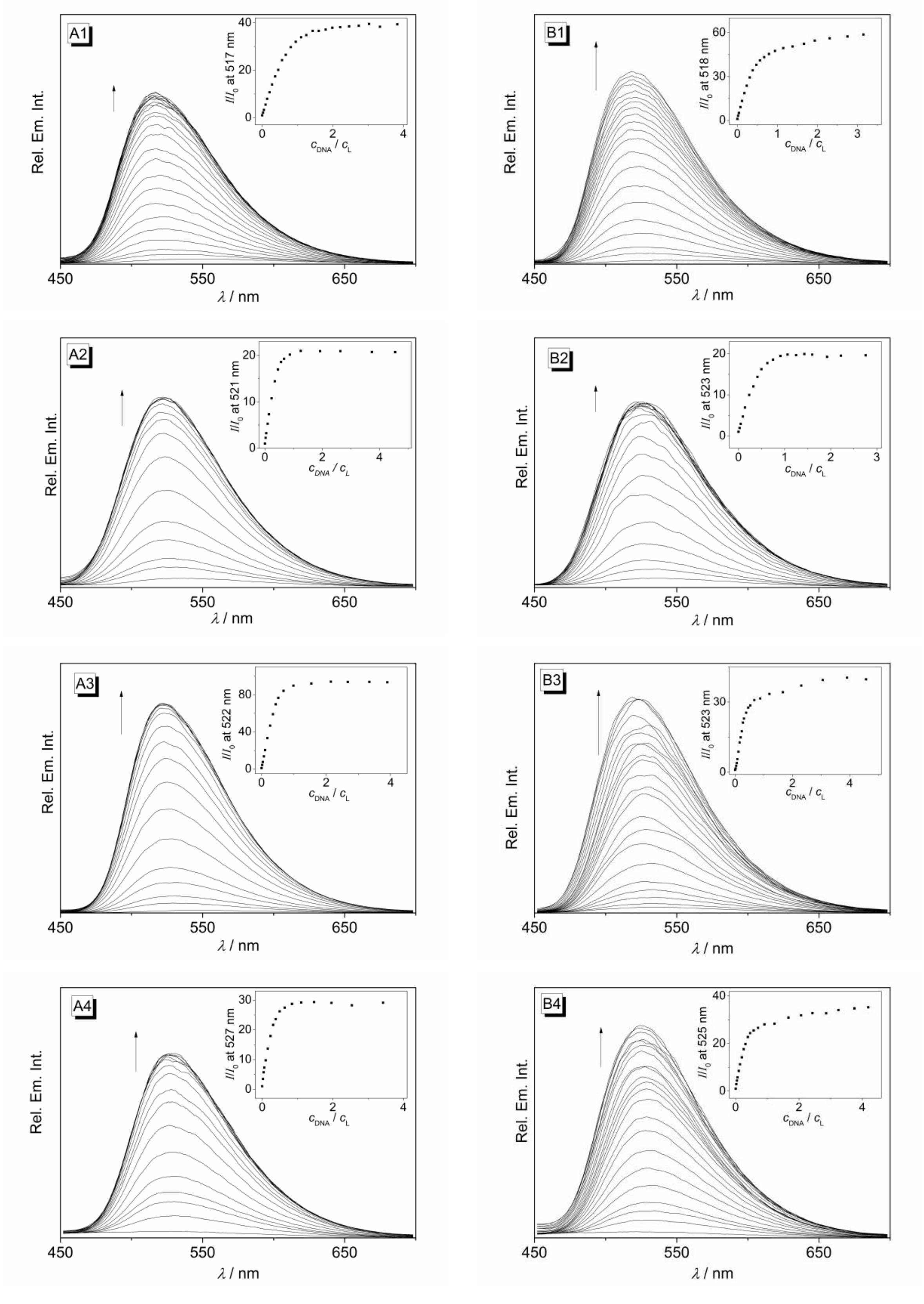

Figure S2. Fluorimetric titration of **4a** (1), **4b** (2), **4d** (3) and **4e** (4) with **22AG** (A) and **a2** (B) (c_L = 20 µM; c_{DNA} = 200 µM) in K^+-phosphate buffer (pH 7.0 with 10% v/v DMSO). The arrows indicate the development of the emission bands with increasing DNA concentration. Inset: Plot of I/I_0 versus c_{DNA}/c_L.

1.1. Determination of binding constants

From the binding isotherms derived from the photometric titrations, the binding constants were determined with SpecFit/32™ (Spectrum Software Associates, PMB 361, 197M Boston Post Road, West Marlborough, MA 01752, U.S.A.). The binding interaction between the ligands **4a–e** and **22AG** and **a2** are treated as a host–guest interaction, where the host molecule (DNA) provides a distinct number of binding sites for the guest molecules (ligand). For the interaction between the ligands and the quadruplex DNA, the following equilibria were considered:

ligand + DNA ⇄ ligand–DNA (1:1 complex)

2 ligand + DNA ⇄ $(\text{ligand})_2$–DNA (2:1 complex)

The experimental values were fitted to the theoretical model by the least square method with a Levenberg–Marquardt algorithm as implemented in the software program. In all cases satisfactory fits were only obtained for 2:1 complexes and/or 1:1 complexes (Figure S3).

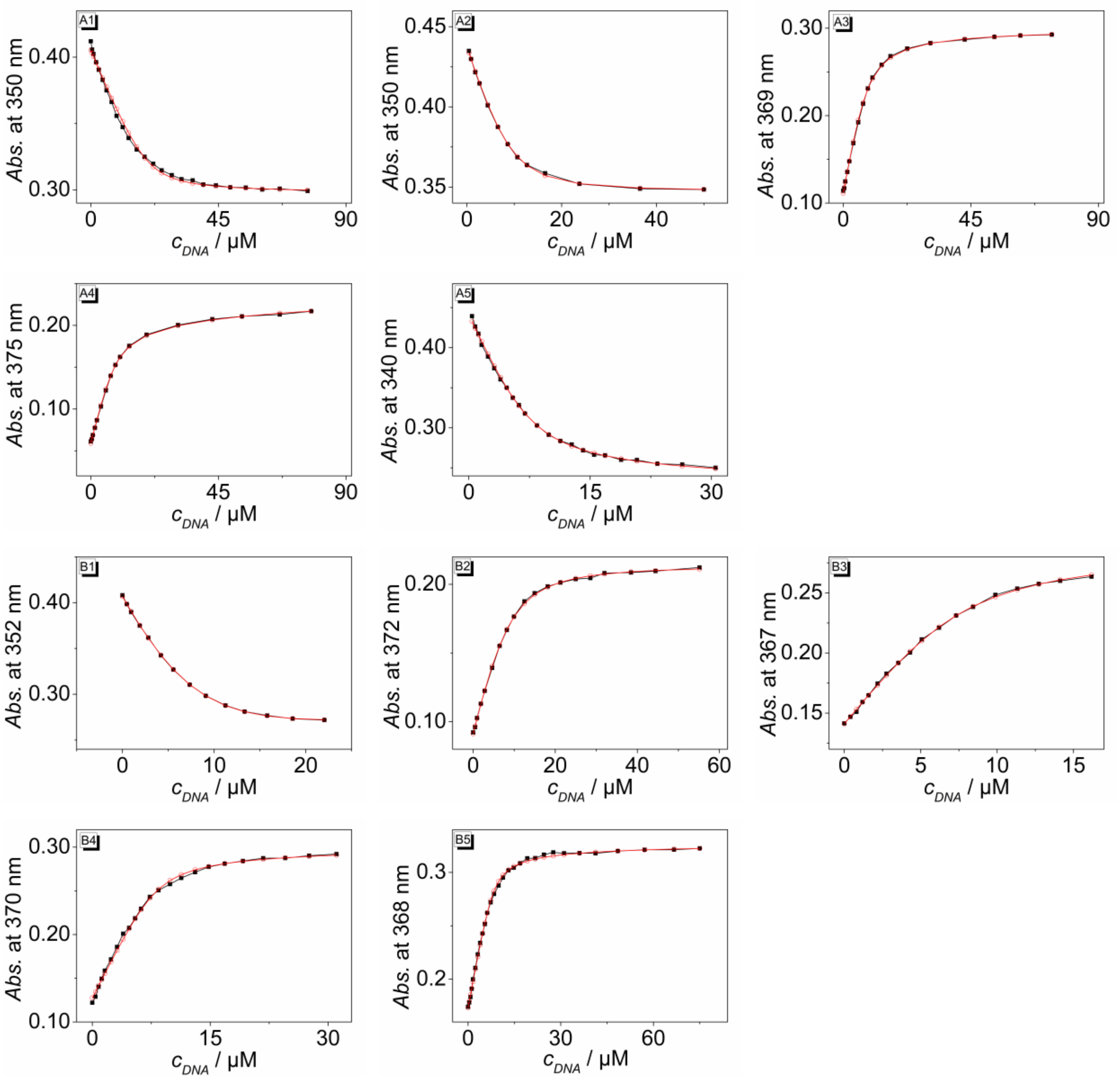

Figure S3. Plot of absorption (*Abs.*) versus concentration of (c_{DNA}) from photometric titrations of G4-DNA **22AG** (A) or **a2** (B) to ligands **4a** (1), **4b** (2), **4c** (3), **4d** (4) and **4e** (5) in K^+-phosphate buffer (pH 7.0 with 10 % v/v DMSO) and fitting of the experimental data to the theoretical model. Black: experimental data; Red: fitting curve.

2. CD spectroscopy

The following parameters were used to collect the CD spectra:

Wavelength range	230–550 nm
Bandwidth	1.0 nm
Scan rate	1.0 nm/s
Time per datapoint	0.5 s
Temperature	20 °C

Table S1. Composition of samples for CD-spectroscopic measurements.

Sample[a]	c_L / µM	V_L / µL[b]	*LDR*
1	0	0	0
2	1.0	2.0	0.05
3	4.0	8.0	0.20
4	10	20	0.50
5	20	40	1.00

[a] c_{DNA} = 20 µM; V_{DNA} = 200 µL; V(buffer) = 1700 µL V(DMSO) = 100 µL; [b] c_L = 1.00 mM in MeOH, solvent was removed prior to the addition of DMSO and buffer.

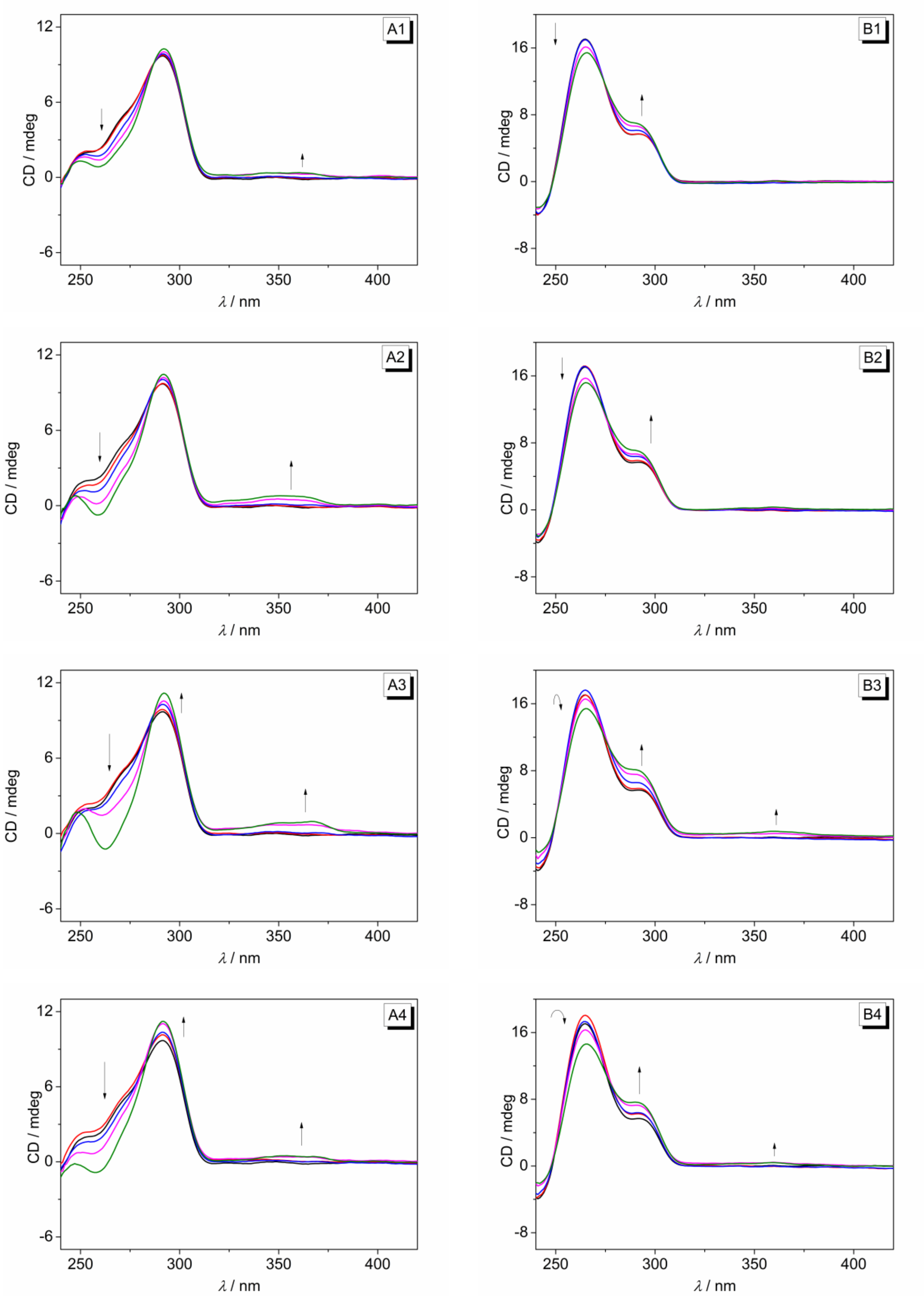

Figure S4. CD spectra of **22AG** (A) and **a2** (B) in the presence of the ligands **4a** (1), **4b** (2), **4d** (3) and **4e** (4) in K^+-phosphate buffer (pH 7.0 with 10% v/v DMSO) at *LDR* = 0.00 (black), 0.05 (red), 0.20 (blue), 0.50 (magenta), and 1.00 (green); c_{DNA} = 20 µM; *T* = 20 °C. The arrows indicate the development of the CD bands with increasing *LDR*.

3. Thermal DNA denaturation

Five solutions of **F21T**, **Fa2T**, **FmycT**, **FkitT**, or **FkrasT** and the ligands **4a**–**e** in cacodylate buffer (10 mM sodium cacodylate, 100 mM LiCl, 90 mM LiCl and 10 mM KCl, pH 7.2) were prepared according to Table S2.

Table S2. Composition of the solutions for the thermal DNA denaturation.

Sample[a]	c_L / µM	V_L / µL[b]	*LDR*	V_{buffer} / µL
1	0.0	0.0	0.0	996.0
2	0.3	12.5	1.25	983.5
3	0.5	25.0	2.50	971.0
4	1.0	50.0	5.00	946.0

[a] c_{DNA} = 0.2 µM; V_{DNA} = 4 µL; [b] c_L = 20 µM.

The excitation wavelength was 470 nm and the change of the emission at 515 nm was detected. During the measurement, the instrument was purged with nitrogen to avoid condensation of water on the cuvette at lower temperatures. The slit width for excitation and emission wavelengths was set to 5 nm and the detector voltage was 600 V. The following temperature program was used:

1) Heating from 20 °C to 90 °C; heating rate: 2.5 °C / min
2) 90 °C for 5 min
3) Cooling to 10 °C, cooling rate: 1.0 °C / min
4) 10 °C for 10 min
5) Heating to 90 °C; heating rate: 0.2 °C / min and detection of the emission

The normalized fluorescence spectra were plotted versus the temperature (Figure S5). The melting temperature was calculated as the maximum of the first derivative of the melting curve determined by the Gaussian function as implemented in the Origin software (Version 8.5.1.). The shift of the melting temperature was calculated as $\Delta T_m = T_m(\text{sample}) - T_m(\text{DNA})$.

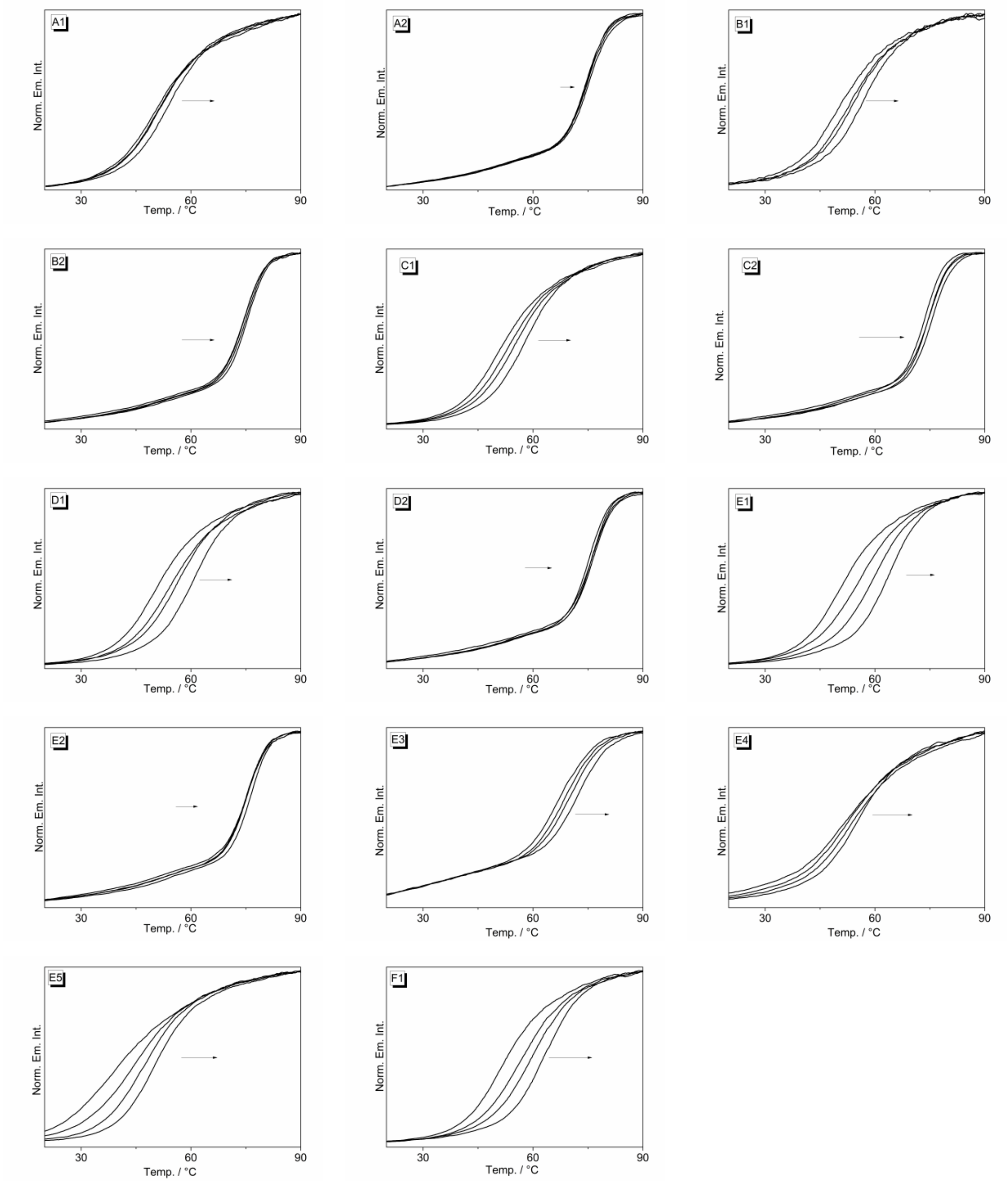

Figure S5. Normalized emission intensities of G4-DNA **F21T** (1), **Fa2T** (2), **FmycT** (3), **FkitT** (4) and **FkrasT** (5) (0.2 µM) (λ_{ex}= 470 nm, λ_{fl} = 515 nm) plotted versus temperature in the presence of ligands **4a** (A), **4b** (B), **4c** (C), **4d** (D) and **4e** (E; F: with **ds26**; c_{ds26} = 3.0 µM) in Na-cacodylate buffer (10 mM K^+, pH 7.2); The arrows indicate the development of the melting curves with increasing *LDR* (0, 1.3, 2.5, and 5.0 molar equivalents).

4. NMR spectra

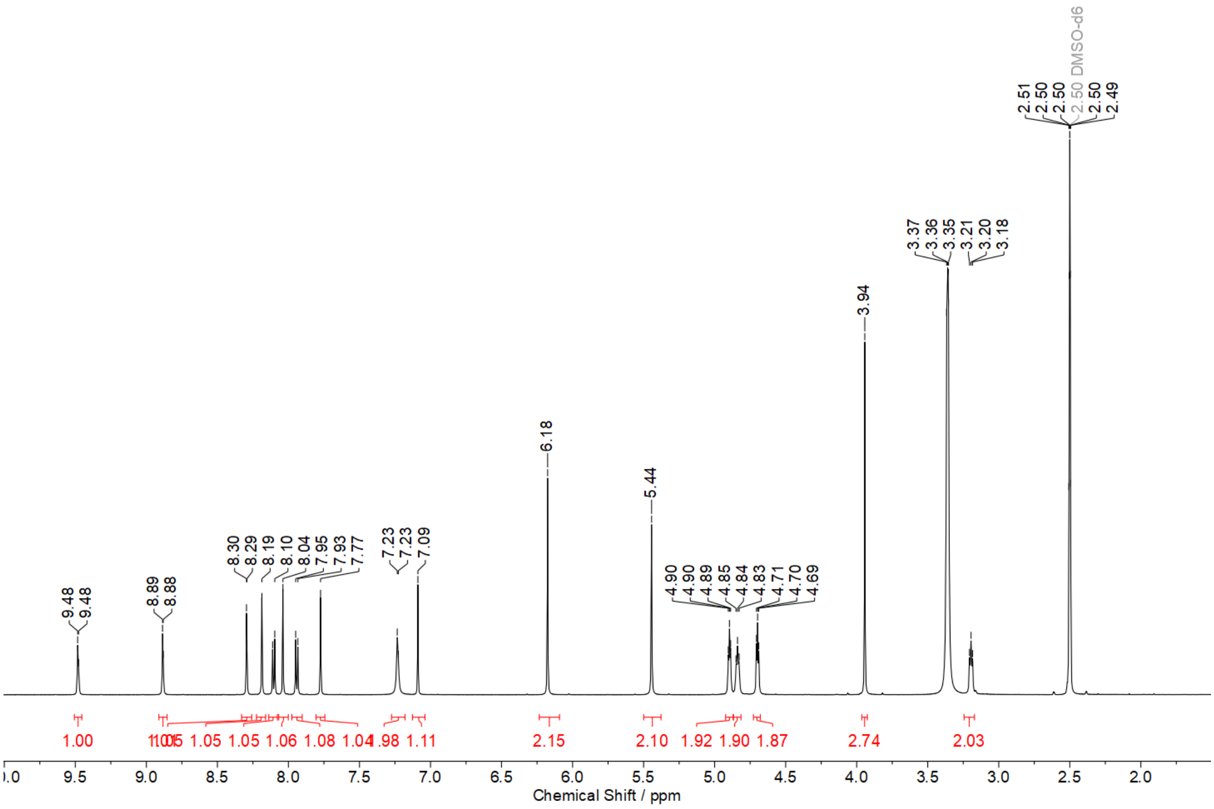

Figure S6. ^{1}H NMR spectrum (600 MHz) of **4a** in DMSO-d_6.

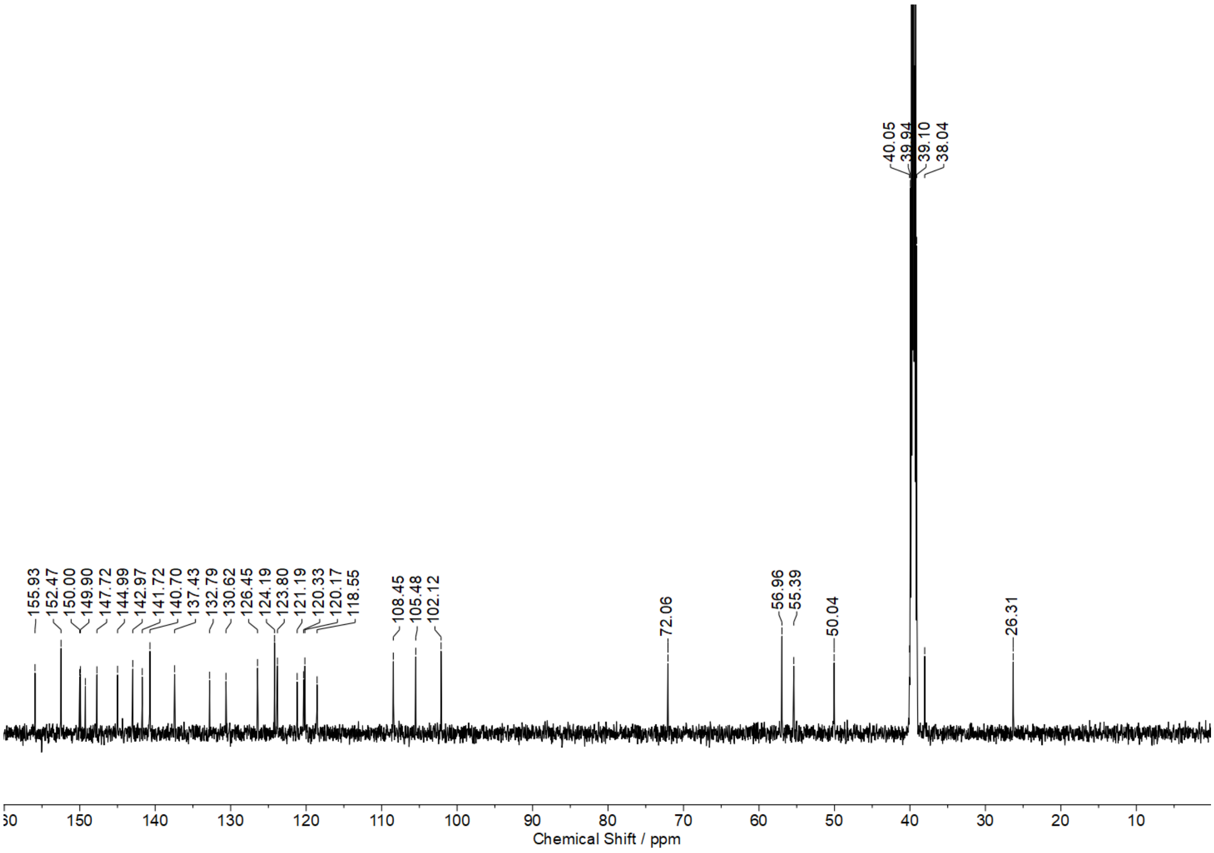

Figure S7. ^{13}C NMR spectrum (150 MHz) of **4a** in DMSO-d_6.

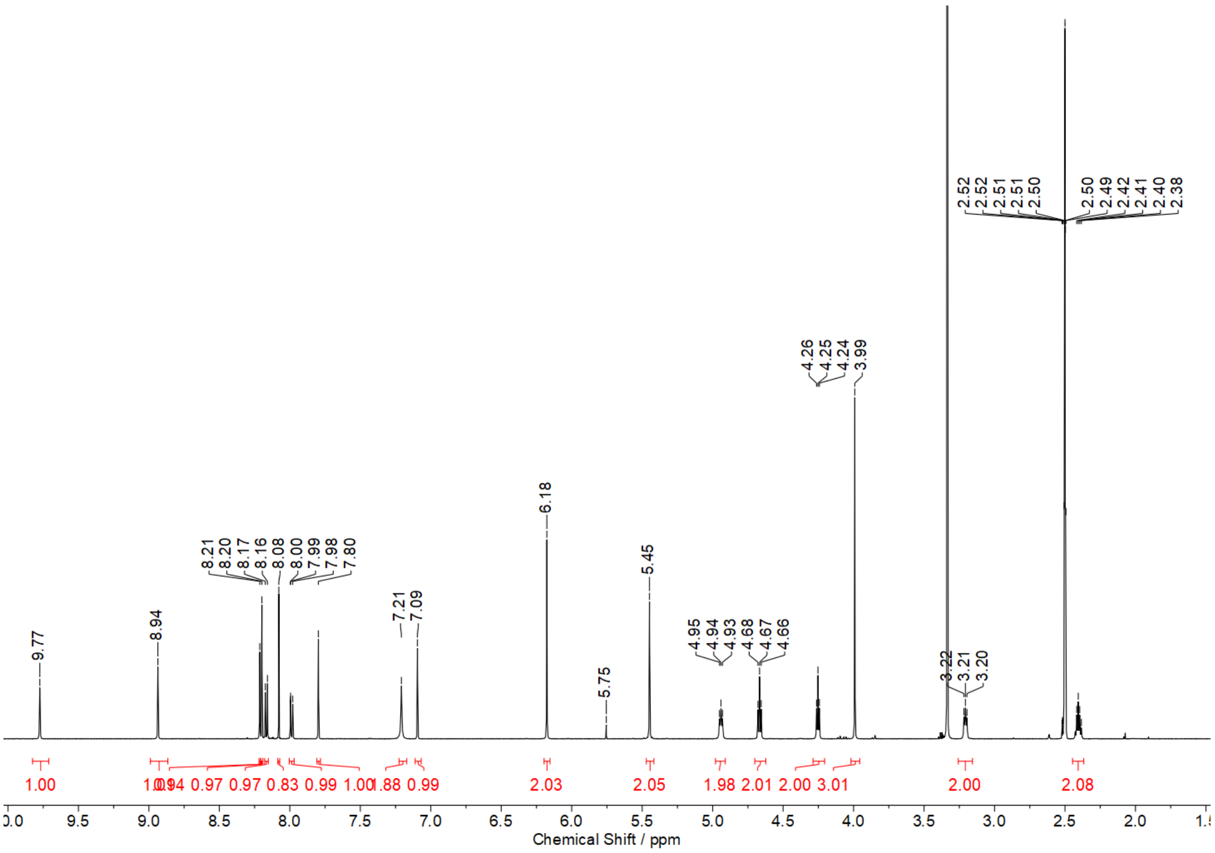

Figure S8. ^{1}H NMR spectrum (600 MHz) of **4b** in DMSO-d_6.

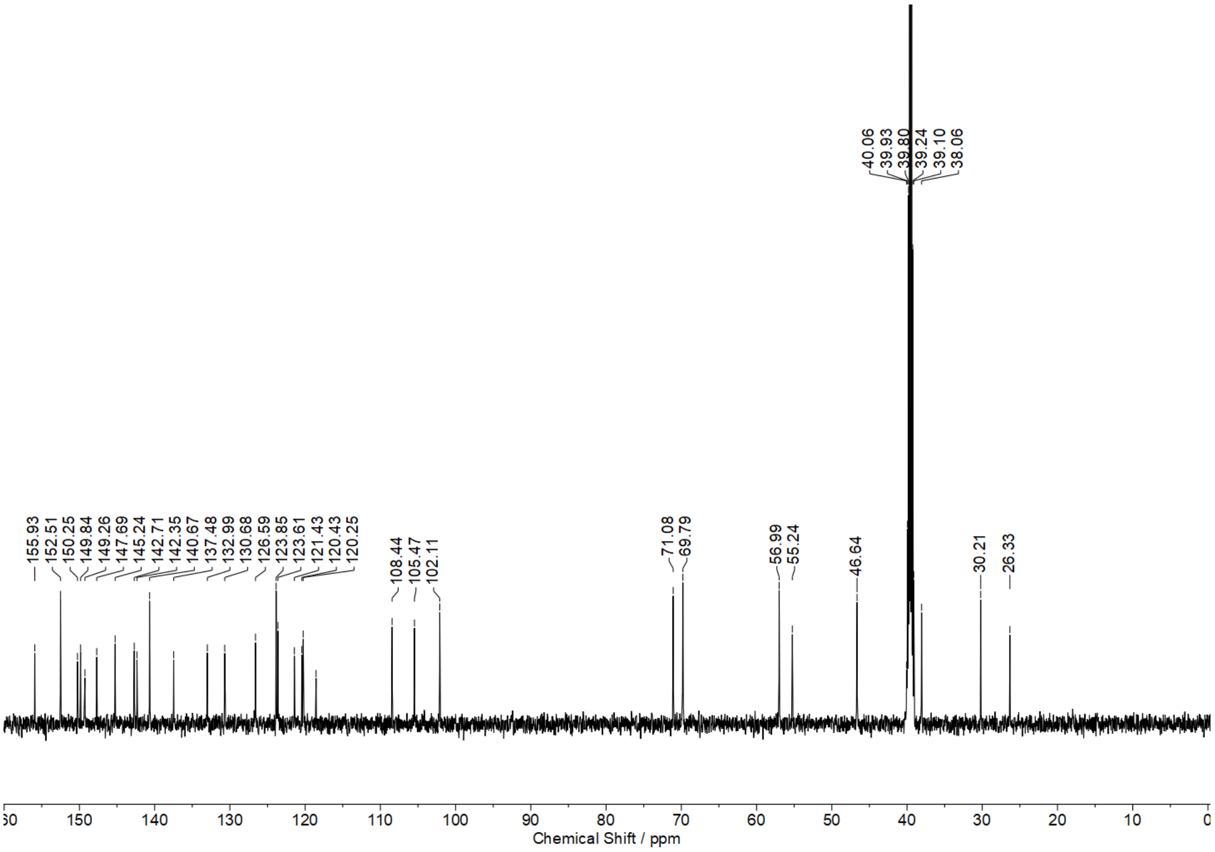

Figure S9. ^{13}C NMR spectrum (150 MHz) of **4b** in DMSO-d_6.

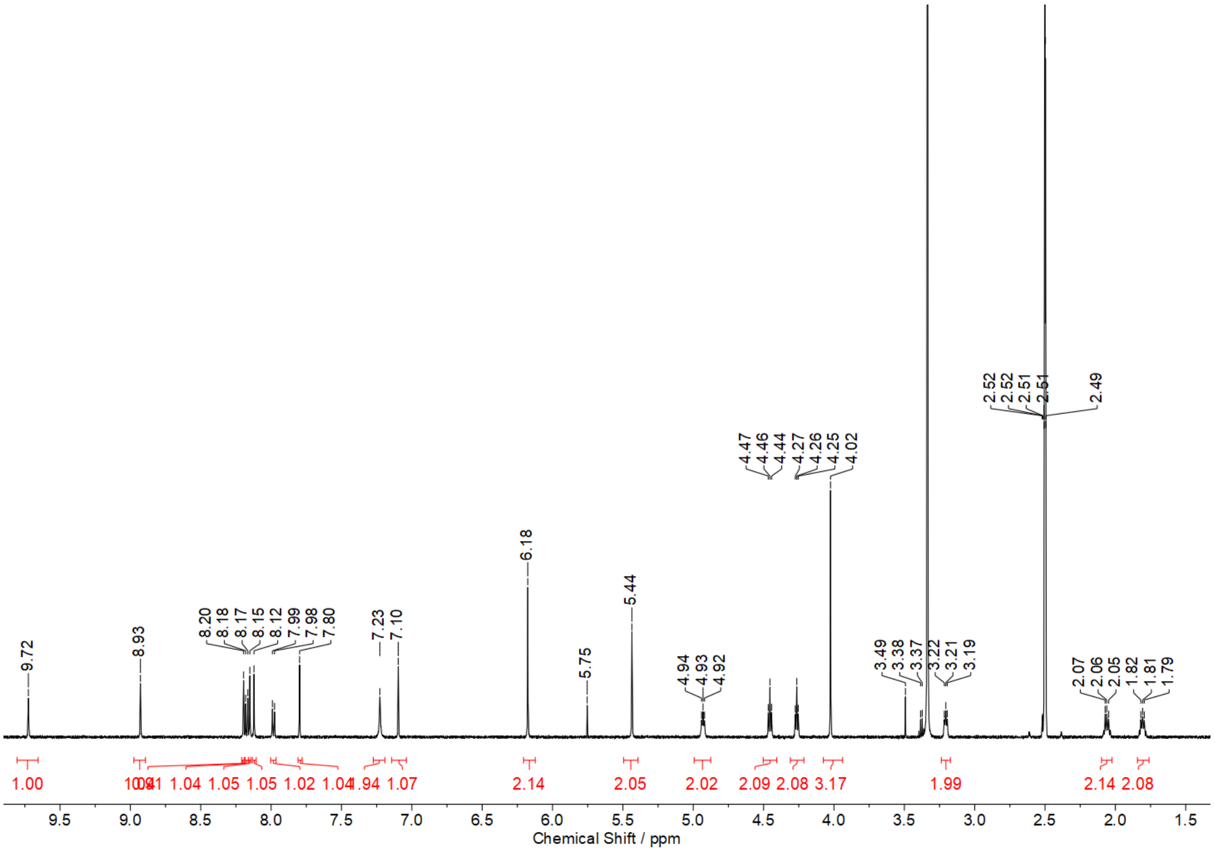

Figure S10. ^{1}H NMR spectrum (600 MHz) of **4c** in DMSO-d_6.

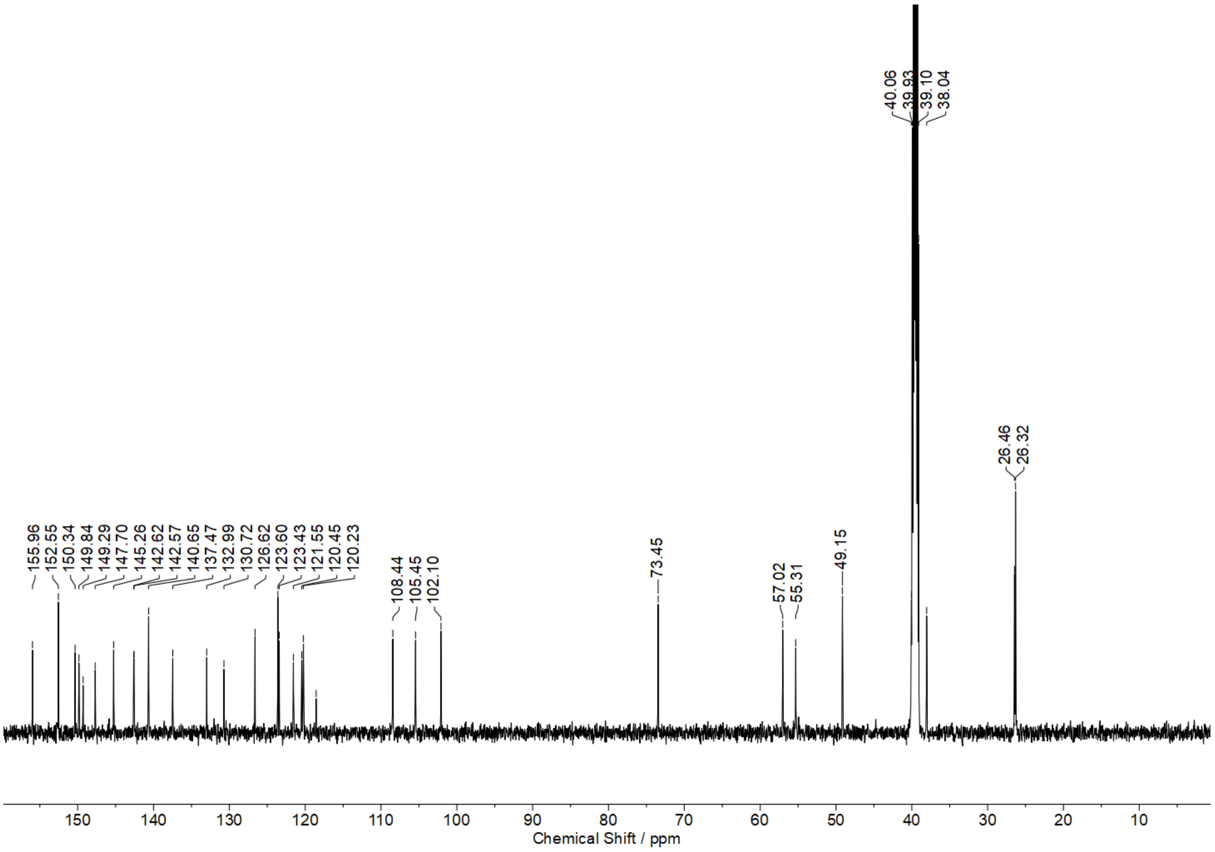

Figure S11. ^{13}C NMR spectrum (150 MHz) of **4c** in DMSO-d_6.

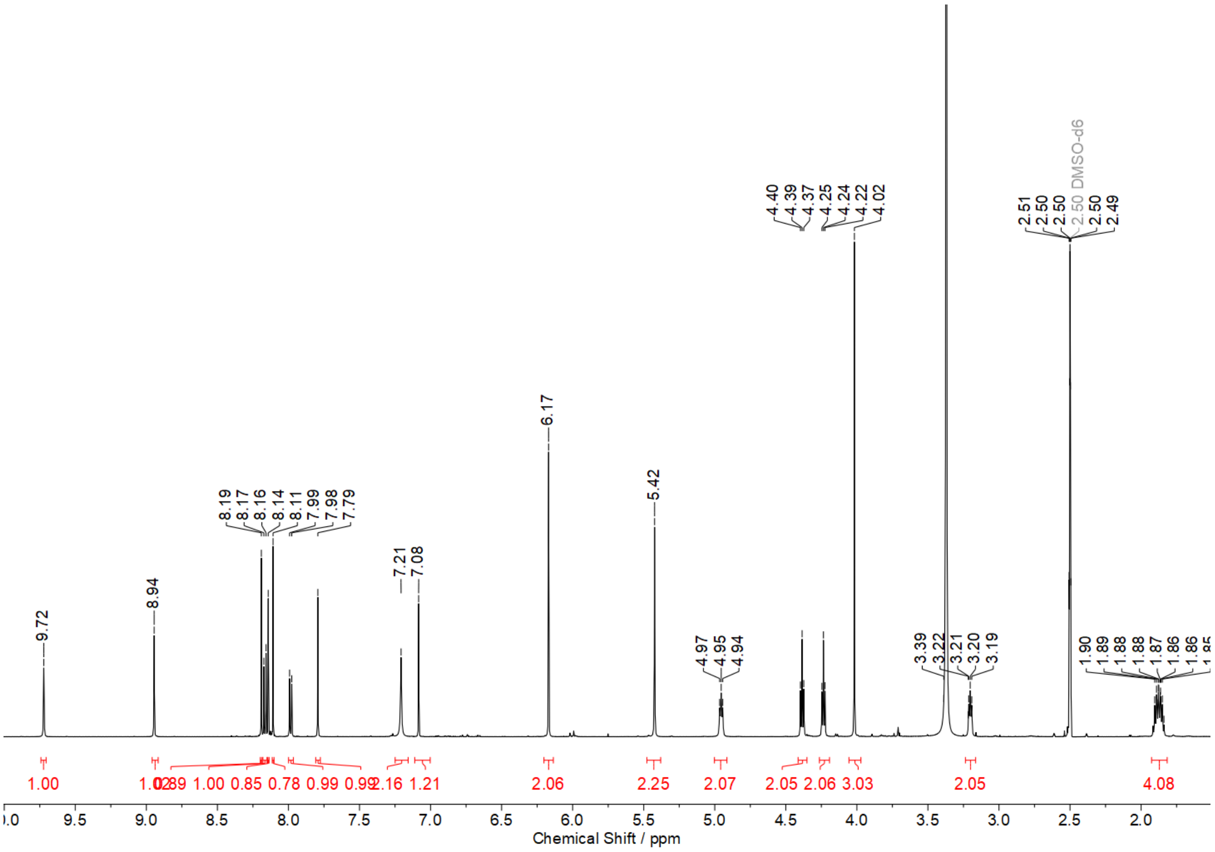

Figure S12. ^{1}H NMR spectrum (600 MHz) of **4d** in DMSO-d_6.

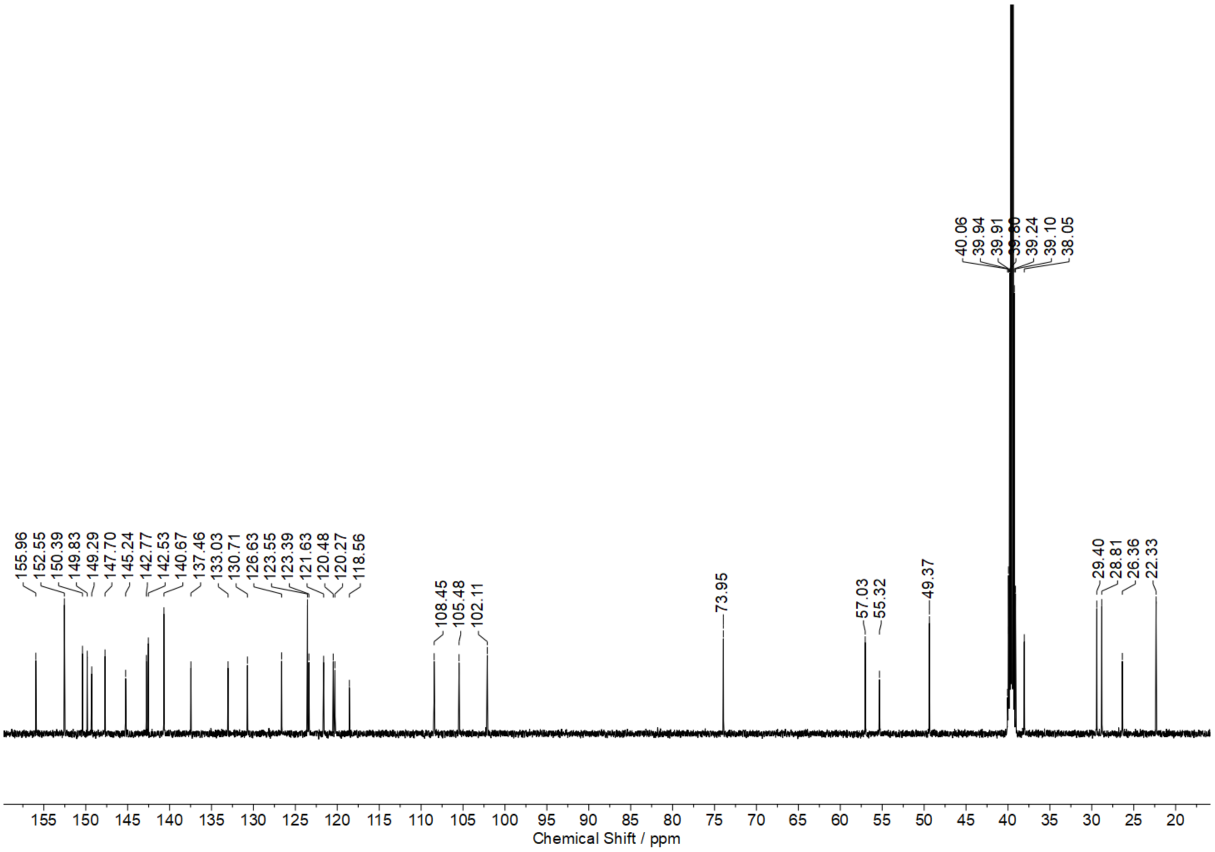

Figure S13. ^{13}C NMR spectrum (150 MHz) of **4d** in DMSO-d_6.

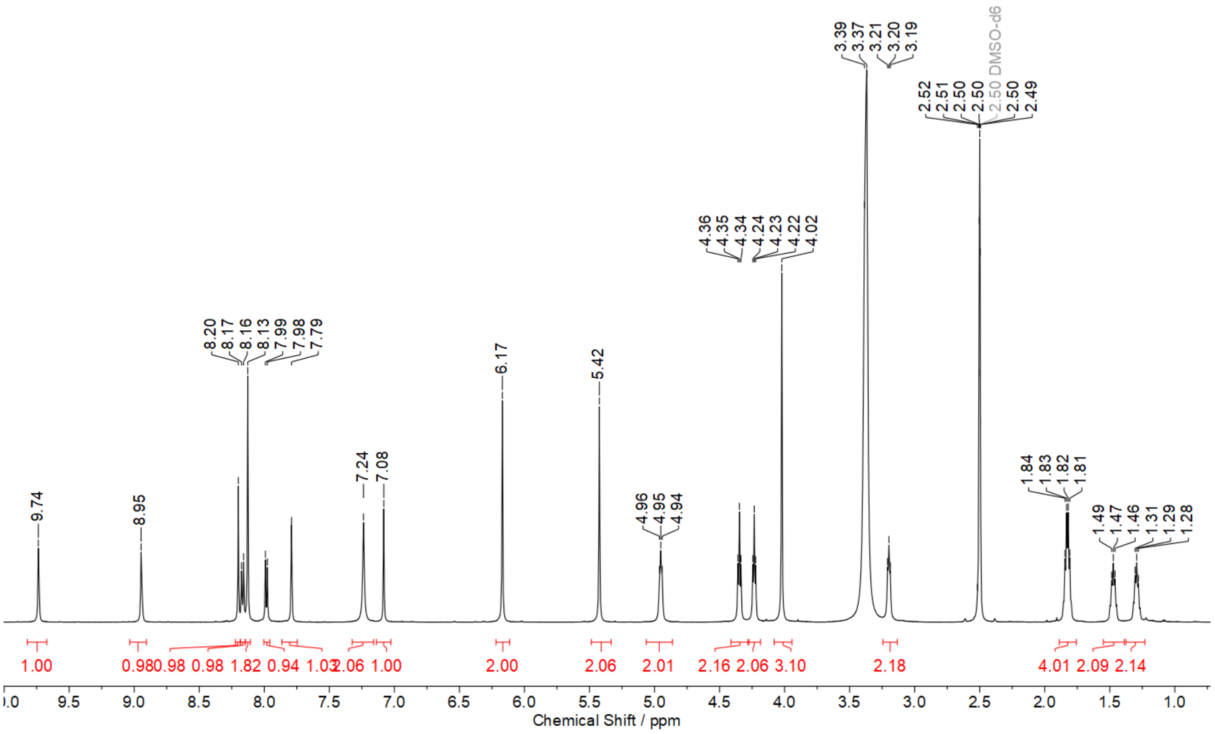

Figure S14. ^{1}H NMR spectrum (600 MHz) of **4e** in DMSO-d_6.

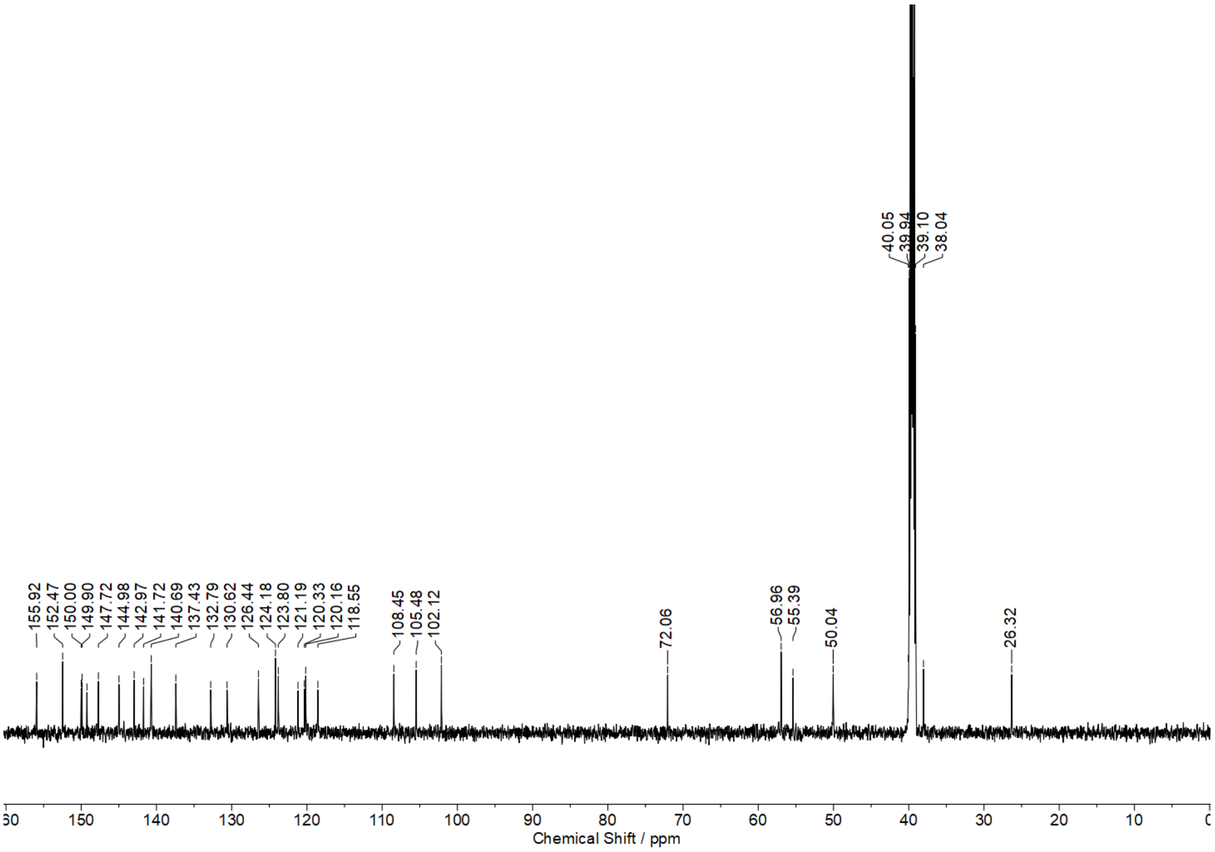

Figure S15. ^{13}C NMR spectrum (150 MHz) of **4e** in DMSO-d_6.
